# New embryological and palaeontological evidence sheds light on the evolution of the archosauromorph ankle

**DOI:** 10.1038/s41598-020-62033-8

**Published:** 2020-03-20

**Authors:** María Victoria Fernandez Blanco, Martín D. Ezcurra, Paula Bona

**Affiliations:** 1 CONICET, Buenos Aires, Argentina; 2 División Paleontología Vertebrados, Museo de La Plata, Paseo del Bosque s/n, 1900 La Plata, Argentina; 3 Sección Paleontología de Vertebrados, CONICET-Museo Argentino de Ciencias Naturales ‘Bernardino Rivadavia’, Avenida Ángel Gallardo 470, C1405DJR Buenos Aires, Argentina

**Keywords:** Embryology, Palaeontology

## Abstract

The homology and evolution of the archosaur ankle is a controversial topic that has been deeply studied using evidence from both extinct and extant taxa. In early stem archosaurs, the astragalus and calcaneum form the ancestral proximal tarsus and a single ossification composes the centrale series. In more recent stem archosaurs, the centrale is incorporated to the proximal row of tarsals laterally contacting the astragalus. This bone is subsequently lost as an independent ossification before the last common ancestor of birds and crocodilians, but the evolutionary fate of this element remains mostly unexplored. Here, we integrate embryological and palaeontological data with morphogeometric analyses to test the hypothesis of loss of the centrale or, alternatively, its incorporation into the archosaurian astragalus. Our results support the latter hypothesis, indicating that the astragalus developed ancestrally from two ossification centres in stem archosaurs and that the supposed tibiale of bird embryos represents a centrale. This conclusion agrees with previous embryological studies that concluded that the tibiale never develops in diapsids.

## Introduction

The tarsus of archosaurs – crocodilians, birds, non-avian dinosaurs and several other extinct clades – and their most immediate precursors has been one of the most deeply studied anatomical regions of this clade because of its strong phylogenetic signal and morphofunctional importance^1–5^. In particular, the homology of the primordial cartilages and their subsequent ossifications that form the archosaur tarsus has been a widely discussed topic that integrates palaeontological and embryological studies^4,6–12^. The proximal tarsus of archosaurs is ancestrally composed of a medial astragalus that articulates proximally with the tibia and fibula and a lateral calcaneum that articulates proximally with the fibula^3,13^. Separate astragalar and calcaneal ossifications have been retained in Crocodylia, but both bones fuse into a single proximal tarsal in Aves^7^. During the embryological development of extant crocodilians, the chondrogenic proximal tarsus possesses three condensations – the intermedium, centrale and fibulare – that subsequently ossify into two elements – the astragalus and calcaneum –^10,11^.

A single ossification originating from the four elements that are proposed to compose the centrale series of amniotes (e.g. juveniles of captorhinids, such as *Moradisaurus grandis*^14^) is retained in stem archosaurs^5^. This single centrale is located immediately mediodistal to the astragalus in the earliest archosauromorphs (e.g. *Protorosaurus speneri*^15^) but is incorporated to the proximal row of tarsals, laterally contacting the astragalus, in more crownward stem archosaurs^16^. As a result, in the latter taxa, the centrale, together with the astragalus, forms part of the articular facet for the distal end of tibia. In archosauromorphs more closely related to Archosauria, the centrale is lost as an independent ossification^5,16^. The absence of a centrale has long been recognized as a synapomorphy within stem Archosauria^4,5,16^, but the evolutionary fate of this element has received relatively poor attention. Some authors have proposed that the centrale fuses with the astragalus to form a single medial proximal tarsal^12,17^. However, this hypothesis remains untested. Here, we integrate embryological and palaeontological data and quantitative methodologies to test the hypothesis of fusion between the centrale and astragalus, or the alternative hypothesis of a complete loss of this element. The potential recognition of the centrale as a component of the proximal tarsus of archosaurs has interesting evolutionary implications in the discussion of the homology of the primordial cartilages that form the astragalus in amniotes and the homology of the cartilaginous condensations of bird embryos.

## Results

### Embryological development of the tarsus of *Caiman*

**Figure 1:**
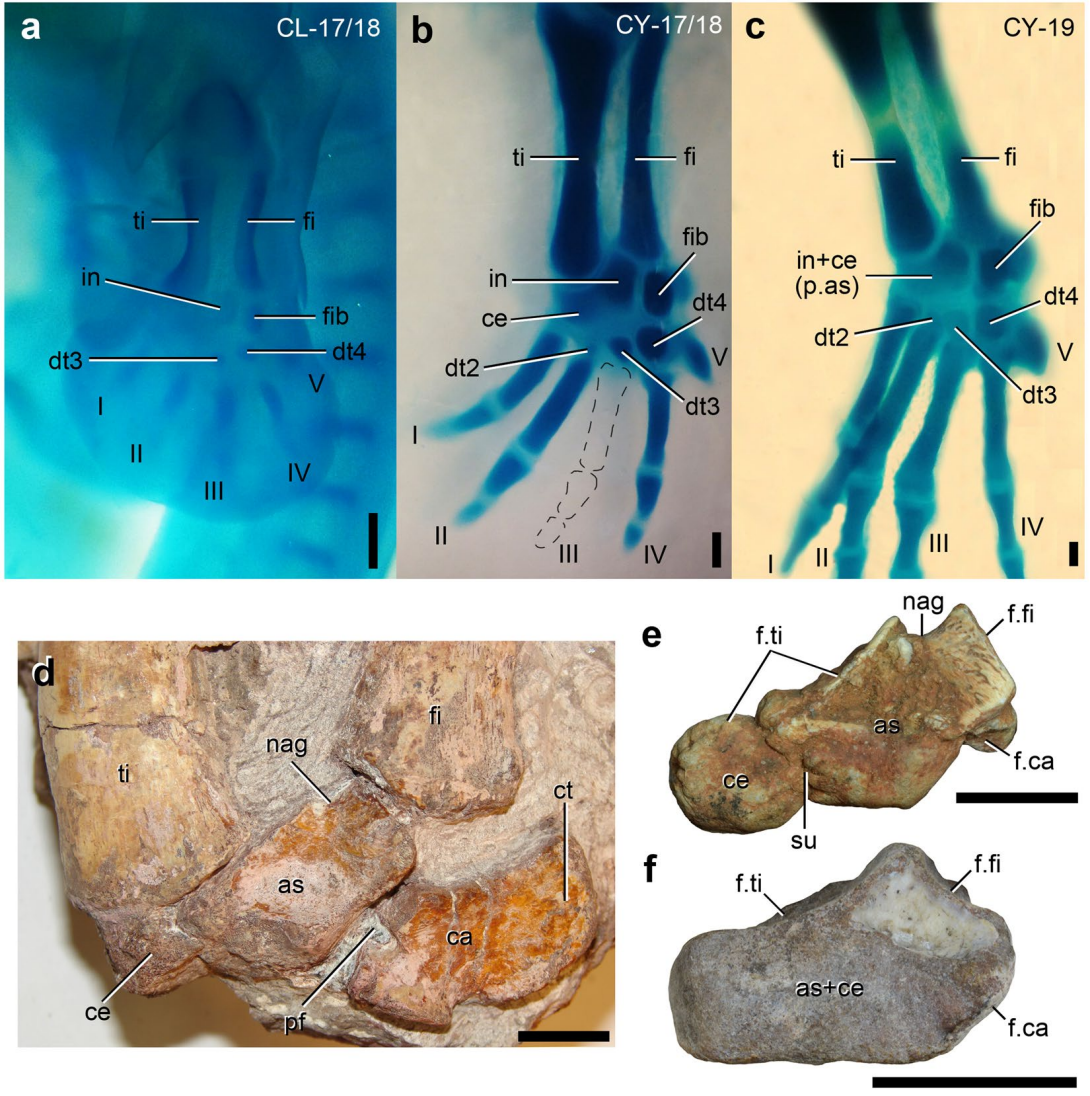
(**a**–**c**) Photographs of embryos of (**a**) *Caiman latirostris* and (**b**,**c**) *Caiman yacare* showing the chondrifications of the tarsus during embryological development; and (**d–f**) astragali and centrale of the Triassic non-archosaurian archosauromorphs (**d**) *Proterosuchus* sp. (AMNH FR 2237), (**e**) *Pamelaria dolichotrachela* (ISIR 316) and (**f**) a Rhadinosuchinae indet. (CRILAR-Pv 492) in anterior views. Abbreviations: I–V, digits I–V; as, astragalus; ca, calcaneum; ce, centrale; ct, calcaneal tuber; dt2–4, distal tarsals 2–4; in, intermedium; f.ca, facet for calcaneum; f.fi, facet for fibula; f.ti, facet for tibia; fi, fibula; fib, fibulare; nag, non-articulating gap; p.as, precursor of the astragalus; pf, perforating foramen; su, suture; ti, tibia. Scale bars equal 0.2 mm in (**a–c**), 1 cm in (**d**) and 5 mm in (**e**,**f**). Photographs (**b**,**d**,**f**) reversed.

In the earliest-sampled embryonic growth stages of *Caiman yacare* [CY] and *Caiman latirostris* [CL] (MLP-R.6491 CL-17/18 and MLP-R.6490 CY-17/18, see Materials and Methods for nomenclature of growth stages) there are two cartilaginous condensations that articulate proximally and proximolaterally with the distal end of tibia (Fig. 1a–c), resembling the condition in bird embryos^18^. A single thin layer of cartilage extends over the proximal surface of both condensations and contributes to the tibial articulation (MLP-R.6490 CY-17/18-1). We interpret these condensations as the intermedium laterally and the centrale medially. The proximal surface of the intermedium articulates with the lateral and medial halves of the distal end of tibia and fibula, respectively. The lateral surface of the intermedium articulates with the fibulare and there is a thin layer of cartilage that will participates in the future crurotarsal joint. This same layer extends distally to form the articulation between the fibulare and a large distal tarsal 4. The distal tarsal 3 is present medial to the distal tarsal 4 and proximal to the metatarsal III, and immediately proximal to metatarsal II there is a small, spherical condensation that we identify as distal tarsal 2. In the later embryonic stage (MLP-R.6490 CY-19) there is no differentiation between an intermedium and centrale, which creates a single centre of ossification, the astragalus, in more advanced embryos. In all sampled stages of both species we have not observed a segmentation or a condensation adjacent to the distal end of the tibia that may indicate the development of a tibiale, in agreement with the absence of this element in at least several diapsids – including turtles^19^ – (e.g. refs. ^11,12,20^).

### Evolution of the tarsus in early archosauromorphs

In the ancestral condition of the archosauromorph tarsus, the centrale is located ventral to the medial half of the astragalus and, as a result, it does not articulate with the tibia. In more crownward archosauromorphs (Crocopoda), the centrale occupies a proximal position in the tarsus and is located medial to the astragalus, participating in the articulation with the tibia^4,5,12,16^ (Fig. 1d,e). In addition, the centrale fuses with the astragalus in some individuals of allokotosaurs, rhynchosaurs and proterosuchids^17,21^. All known specimens of the azendohsaurid allokotosaurs *Pamelaria dolichotrachela* (ISIR 316/58), *Azendohsaurus madagaskarensis*^17^ and *Shringasaurus indicus*^21^ present such fusion, but in the trilophosaurid allokotosaur *Trilophosaurus buettneri* its presence is polymorphic and apparently not related to the size of the individuals^17^. The astragalus and centrale are separated from each other in Early and Middle Triassic rhynchosaurs^22–25^, but the co-ossification between both bones occurs in large-sized individuals of at least some hyperodapedontine rhynchosaurs^17^. Among the Proterosuchidae, a small-sized and probably immature individual of *Proterosuchus fergusi* (SAM-PK-K140^26^) possesses an astragalus unfused to the centrale (the latter bone is not preserved in this specimen). By contrast, the astragalus is co-ossified with the centrale in larger specimens of the genus (e.g. *Proterosuchus sp*.: AMNH FR 2237; *Proterosuchus alexanderi*: NMQR 1484). Thus, at least in proterosuchids, it is possible that the fusion between the centrale and astragalus occurs later in post-hatching ontogeny. In those crocopods, in which the centrale is fused to the astragalus, a line of suture or ventral cleft persists showing the original separation between both elements (Fig. 1d,e). In Erythrosuchidae and Eucrocopoda, the centrale is absent as a separate ossification and the tibial-tarsal facet retains a similar proportional size, although it is formed only by the astragalus (Fig. 1f).

### Analyses of the morphogeometric configurations

**Figure 2:**
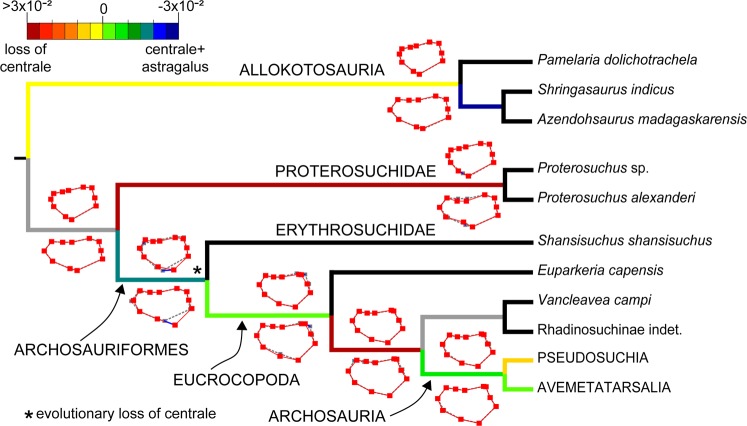
Optimization of both morphogeometric configurations shown in a reduced phylogeny of early archosauromorphs (after Sengupta *et al*.^22^). Colours in the branches indicate the difference in the number of steps between both configurations for the landmarks that sample the medial margin of the astragalus and/or centrale (landmarks 2, 6 and 9, and semi-landmarks 1 and 2). Recovered hypothetical ancestral configurations (red lines) are shown above (only astragalus) and below (astragalus + centrale) each branch. Dotted grey lines show the configuration ancestral to that node and the blue lines indicate the displacement of the landmarks in the ancestor-descendant transformation.

The optimization of the two alternative morphogeometric configurations (sampling only the astragalus or the astragalus + centrale) in the phylogenetic supertrees (see Materials and Methods) recovers the transformation of an astragalus + centrale into the astragalus as more parsimonious than the alternative hypothesis of loss of the centrale (taking into account the length of the branch along which the loss of a separate centrale is optimized) (Fig. 2; Table 1). The main landmark transformation in this branch (i.e. Erythrosuchidae + Eucrocopoda) corresponds to a lateral displacement of the ventral margin of the calcaneal facet (Landmark 8) in both trees (Supplementary Information 1, Supplementary Table S3). This change is related to a shallower notch that receives the ventral portion of the astragalar facet of the calcaneum, which in turn probably corresponds to the loss of the foramen for the passage of the perforating artery in the Erythrosuchidae + Eucrocopoda branch^16^. In the trees optimizing the astragalus + centrale, other changes in landmark and semilandmark positions are considerably lower and the transformations of the landmarks that describe the shape of the medial margin of the structure (i.e. Landmark 2 and semilandmark 2) are very minor. By contrast, in the other configuration, there is a distinct ventromedial displacement of Landmark 2 and a lower, but conspicuous, dorsolateral displacement of semilandmark 2. These changes in the latter optimization show that a medial expansion of the astragalus is the most parsimonious transformation in this branch. However, in the other configuration (astragalus + centrale), the medial expansion of the astragalus is not necessary because this space is occupied by the centrale in more basal branches.

**Table 1:** Results of the optimization of the two alternative morphogeometric configurations in the two phylogenetic topologies of early archosauromorphs.

Tree and configuration	Tree length	CI	Branch length for medial LMs
Ezcurra; astragalus + centrale	6.87994	0.1964	**0.02419**
Ezcurra; astragalus	6.37848	0.2079	0.04097
Nesbitt; astragalus + centrale	6.91867	0.1953	**0.02419**
Nesbitt; astragalus	6.40126	0.2071	0.02459

The scores of branch length are those for the five landmarks that sample the medial margin of the proximal tarsus (landmarks 2, 6 and 9, and semi-landmarks 1 and 2; Supplementary Information 1). Most parsimonious length indicated in bold. Abbreviations: CI, consistency index; LMs, landmarks.

In the Principal Component Analysis, the early archosauromorphs that retain a distinct centrale (i.e. allokotosaurs and proterosuchids) occur at the bottom of the right side of the morphospace (Supplementary Fig. 4). In this quadrant, the early archosauromorphs that are more distant to the region occupied by members of the Erythrosuchidae + Eucrocopoda clade are those for which the centrale was not sampled in the morphogeometric configuration. By contrast, when both astragalus and centrale are sampled together, these taxa occupy a position closer to more derived archosauromorphs and the centroid of the morphospace. In agreement, the Sum of Variances (SoV) of the combined dataset found a lower disparity (SoV = 0.03135668 versus 0.03382903) when the astragalus of erythrosuchids + eucrocopods incorporates the centrale of more basal archosauromorphs.

## Discussion

### The evolutionary fate of the archosauromorph centrale

Some previous authors have interpreted that the medial proximal tarsal of Archosauria is a result of the fusion between the centrale and astragalus that occur as separate ossifications in post-hatching individuals ancestrally in Archosauromorpha (e.g. refs. ^5,12,17^). The results recovered from the optimization of the morphogeometric configurations in the phylogenies and Principal Component Analyses are the first quantitative evidences that support the hypothesis that the centrale has actually fused to the astragalus to form a single ossification in the Erythrosuchidae + Eucrocopoda clade rather than that the centrale failed to ossify. The hypothesis of fusion between the astragalus and centrale agrees with the retention of a suture line in the co-ossified elements that form the medial proximal tarsal of allokotosaurs, some hyperodapedontine rhynchosaurs and medium- to large-sized proterosuchid specimens. The astragalus-centrale fusion also matches the topological relationship and subsequent fusion between the two chondrogenic condensations during the embryological development of the medial proximal tarsal in crocodilians (intermedium and centrale; e.g. *Caiman yacare* and *Caiman latirostris*: this study; *Alligator mississippiensis*^11^; *Melanosuchus niger*^27^) and birds (intermedium and tibiale, e.g. ref. ^18^; but see below). The fusion between the astragalus and centrale occurs during the post-hatching ontogeny in proterosuchid archosauriforms, but the merging of the structures that we consider as equivalent (i.e. intermedium and centrale) takes place during the stage of cartilaginous condensation in the tarsus of crocodilian and bird embryos. This results in a single ossification centre that forms the definitive astragalus of extant archosaurs. As a consequence, we interpret that a peramorphic heterochronic event in the timing of fusion between the components of the medial proximal tarsal should have occurred in the branch leading to the Erythrosuchidae + Eucrocopoda clade.

### The centrale and tibiale as components of the astragalus in archosauromorphs

Multiple embryological studies have shown that there are two chondrogenic condensations that form the single ossification centre that constitutes the astragalus of crocodilians and birds. In particular, Ossa-Fuentes *et al*.^18^ have recently found compelling evidence that the intermedium forms the ascending process of the astragalus (a structure absent in crocodilians and synapomorphic for Dinosauriformes^28^) and another chondrogenic condensation forms the astragalar body during the embryological development of birds. These authors identified this latter condensation as a tibiale following previous embryological studies of birds, although they acknowledge that other authors have proposed the absence of a tibiale in reptiles and that this cartilage was alternatively interpreted as a proximal centrale^10,11^. If a tibiale homologous to the ossified tibiale of early tetrapods is present in bird embryos it would contradict the hypothesis supported here of the incorporation of the centrale to the astragalus –to form a single centre of ossification– in early archosaurs and their immediate precursors (e.g. *Euparkeria*, proterochampsids).

The tibiale develops from a transverse segmentation of the distal end of the tibia in non-amniote tetrapods^9–11^ and embryological studies on reptile limb development have not observed such segmentation and, as a result, proposed that the tibiale never forms in diapsids^9–12,20^. Instead, the element that chondrifies in the equivalent position as the tibiale in diapsid embryos lacks a connection with the distal end of tibia, but it contacts the intermedium and has been therefore interpreted as a centrale^10^. As a consequence, the medialmost chondrification in the proximal tarsus of bird embryos should not be homologous to a tibiale. The evidence presented here for an archosaur astragalus composed of the ancestral centrale and astragalus of diapsids reinforces the interpretation that the two chondrogenic condensations that form the astragalus in birds should be identified as the intermedium and the centrale, as occurs in lepidosauromorph, turtle and crocodilian embryos (e.g. refs. ^11,12,27,29^).

Our interpretations also have implications for the homology of the single centrale ossification retained in archosauromorphs. The separate chondrogenic condensation of the most preaxial centrale (centrale 1) originates from a segmentation of the tibiale in non-amniote tetrapods (e.g. *Ambystoma*^10^). Thus, the absence of a tibiale in Diapsida implies the absence of such preaxial centrale. Previous authors have interpreted that the intermedium and centrale 4 are integrated to form the amniote astragalus (the three-centre model of Peabody^8^). An alternative hypothesis, the four-centre model, also includes the centrale 3 as part of the amniote astragalus^14^. As a consequence, following these evolutionary hypotheses that the centrale 4, and possibly the centrale 3 as well, form part of the astragalus, the single independent centrale ossification retained in archosauromorphs should be homologous to the centrale 2 (given the four-centre model) or centrale 2 + 3 (given the three-centre model) of non-amniote tetrapods. This interpretation contradicts the traditional hypothesis that the centrale that chondrifies in the tarsus of diapsid embryos is homologous to the centrale 4, a view often followed by embryological studies (e.g. refs. ^27,29^). In addition, the centrale of crocodilian embryos and early archosauromorphs shows a topological relationship that matches the position of centrale 2 in early tetrapods^14^.

### The evolution of the developmental pathway of the archosauromorph astragalus

**Figure 3:**
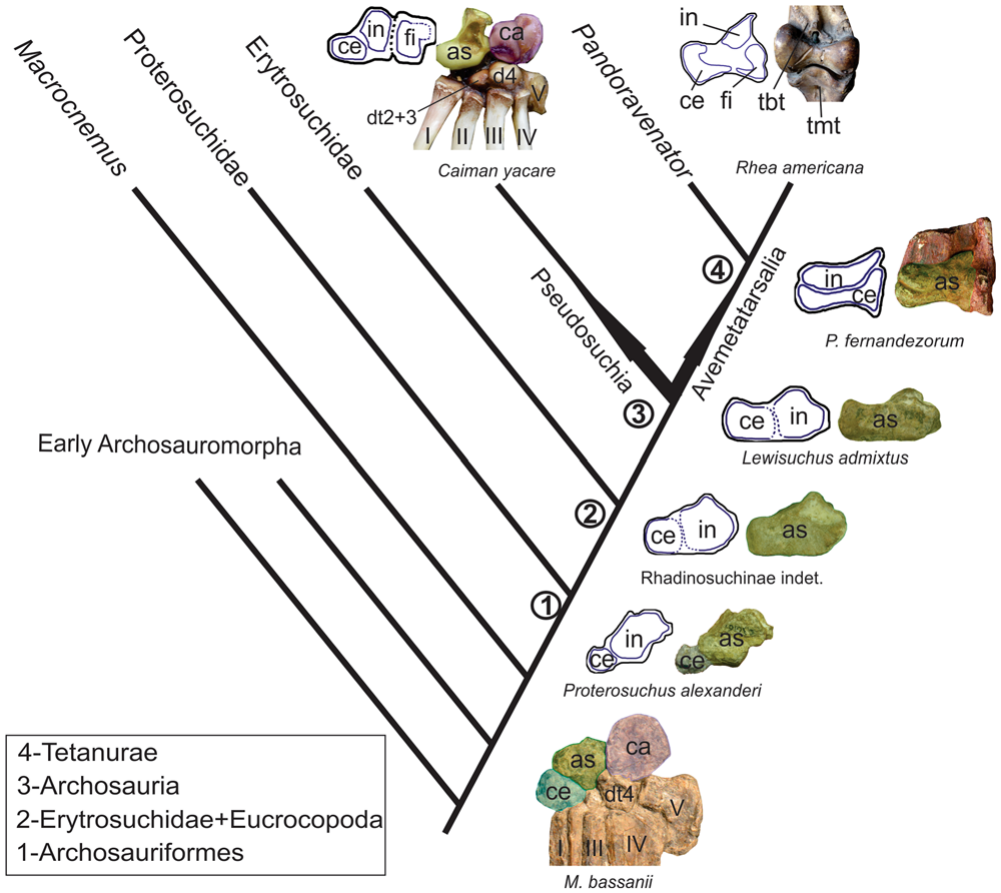
Simplified cladogram of Archosauromorpha (after Ezcurra^17^) showing the evolution of the proximal tarsus hypothesized here. Photographs of post-hatching specimens in anterior view (right) and outline drawings of cartilaginous condensations in extant species as continuous lines (Crocodylia and Aves) or hypothetical condensations as dotted lines for fossil species (left). In all cases lateral is to the right of the figure. Abbreviations: I–V, metatarsals I–V; as, astragalus; asp, ascending process; ca, calcaneum; ce, centrale; d4, distal tarsal 4; fi, fibulare; in, intermedium; tbt, tibiotarsus; tmt; tarsometatarsus. Collection numbers: *Macrocnemus bassanii* (PIMUZ T4355); *Proterosuchus alexanderi* (NMQR 1484); Rhadinosuchinae indet. (CRILAR-Pv 492); *Lewisuchus admixtus* (MACN-Pv 18954); *Pandroravenator fernandezorum* (MPEF-PV 1733-4).

The results obtained here allow hypothesizing about the developmental pathways of the archosauromorph astragalus. The centrale of early archosauromorphs forms the medial one-third of the proximal tarsus. As a result, we infer that the centrale should also have been medially restricted in the embryos of these extinct species (Fig. 3). By contrast, the homologous chondrogenic condensations in crocodilian embryos (centrale and intermedium) form each approximately half of the medial proximal tarsal. Thus, it could be inferred that during the evolution towards extant crocodilian species a modification of the developmental pathway occurred in which the centrale increased its proportional participation in the composition of the ossification centre that results in the astragalus. Unfortunately, we are not aware of any fossil pseudosuchian embryo or juvenile that preserves evidence of the size of the separate condensations that constitute the astragalus and, as a consequence, we lack information of how this modification occurred through phylogeny.

It is interesting to note that, at least in the early embryological development of some bird embryos, the intermedium and centrale contribute in an approximately equal amount to the medial proximal tarsal, but this condition changes subsequently in later embryological stages as the centrale comes to form most of the astragalus and the intermedium becomes restricted to the ascending process^18^. Therefore, it could be hypothesized that the ancestral archosaur had a sub-equally developed intermedium and centrale because this is the condition observed in the embryos of both extant birds and crocodilians. The fossil record of theropod dinosaurs preserves evidence of the modifications in the developmental pathway of the astragalus. The early tetanuran *Pandoravenator fernandezorum* and some other basal averostrans possess a well-defined, mostly transverse groove on the anterior surface of the astragalus^30^. This groove subdivides the bone into a dorsomedial portion that includes the ascending process and a ventrolateral one that covers most of the astragalar body^30^. Thus, this condition seems to represent an intermediate stage in which the intermedium was dorsomedially restricted in the astragalus and the centrale was extended substantially laterally and ventrally to the intermedium, from the inferred ancestral archosaur condition, to form most of the astragalar body. A condition closer to that present in late bird embryos is found in some fossil coelurosaurian theropod specimens, in which the subdivision of the astragalus is placed more dorsally than in early averostrans and suggests a more proximally restricted intermedium^19,30^.

## Materials and Methods

### Embryological analysis

Pre-hatching ontogenetic series of 37 embryos of *Caiman latirostris* and 34 of *C. yacare* were studied. Specimens are housed in the herpetological collection of the Museo de La Plata [MLP] (Buenos Aires, Argentina) under a collection number for each ontogenetic series, MLP-R.6490 for *C. yacare* and MLP-R.6491 for *C. latirostris* (Supplementary Table 1). In addition, the growth stage in each ontogenetic series is indicated as CL (*Caiman latirostris*) and CY (*Caiman yacare*) followed by the number of the embryonic stage sensu Iungman *et al*.^31^. The material was collected during three field trips to the Chaco Province in 2012, 2015 and 2018. All the eggs belong to two nests per species removed from nature and were artificially incubated with constant conditions of relative humidity (95%) and temperature (30 °C ± 1). Embryos of both species were collected every day until hatching, fixed and kept in a 5% formaldehyde solution with calcium carbonate. Embryonic stages 20 to 27–28 and 17/18 to 25 (sensu Iungman *et al*.^31^) were sampled in *C. latirostris* and *C. yacare*, respectively. Embryos were prepared for the observation of cartilage and bone according to the double staining and diaphanization technique of Taylor & Van Dyke^32^. Fixed embryos were immersed in baths of successively increasing alcohol concentration, embedded in Alcian Blue, submerged in 1% KOH, inserted in Alizarin red, and finally submerged again in 1% KOH. They were left in KOH solution until the material was totally cleared and the cartilages and bones were visible. Each specimen is conserved in glycerol and was studied using a Zeiss stereomicroscope and a NIKON Stereo Microscope SMZ745/SMZ745T magnifier with a NIKON NI-150 Illuminator illumination. Photographs were taken using a Nikon D40 camera.

We complied with all relevant ethical regulations for animal testing and research. All experimental protocols concerning embryonic manipulation were developed during the PhD research of MVFB and approved by the Facultad de Ciencias Naturales y Museo-Universidad de La Plata, accordingly with the governmental regulations of the Chaco Province.

### Geometric morphometric analysis

The two-dimensional geometric morphometric analysis was conducted on a sample of five astragali and centrale and 37 astragali (in taxa lacking a separate centrale) of Triassic archosauromorphs. The landmarks were placed on photographs of the astragali and centrale in anterior view taken at first hand by one of the authors (MDE), and some other photographs were taken from the literature. The sampled archosauromorph species are housed in different repositories worldwide and are listed in Supplementary Table 2. The shape sample was focused on the outline of the dorsal, ventral and medial margins of the bone and not on the calcaneal articular region. Right tarsals were mirrored in Photoshop CS6 version 13.0 in order to always analyze the same side (left). We selected a series of nine type II and III landmarks^33^ and two semilandmarks (Supplementary Table 3). We used two different morphogeometric configurations of the 42 taxa in order to test the hypothesis about the fate of the centrale bone. The first configuration samples the astragalus and centrale as a single unit to test the hypothesis that the centrale is incorporated to the astragalus to form a unique undivided structure in erythrosuchids and eucrocopods. On the other hand, the second configuration samples only the astragalus to test the alternative hypothesis (Supplementary Fig. 1).

The landmarks and semilandmarks were digitized using the programs tpsUtil 1.76^34^ and tpsDig2 2.31^35^. A Generalized Procrustes analysis was conducted on both configurations, sliding the semilandmarks along their tangent directions using the Procrustes distance criterion, with the function gpagen of the package geomorph 3.0.1^36^ in the software environment R^37^. A Principal Component Analysis was conducted on all the aligned coordinates and the Sum of Variances was calculated for both groups, respectively, as a descriptor of the morphospace. The aligned coordinates of each configuration were exported to TNT 1.5^38^ and they were used as a single morphogeometric continuous character to test each hypothesis. The configurations were re-aligned in TNT applying the minimum distances criterion and optimized using maximum parsimony on two alternative phylogenetic topologies (Supplementary Information 1). In both topologies, the tree was rooted with a clade composed of three allokotosaurian species (*Pamelaria dolichotrachela*, *Shringasaurus indicus* and *Azendohsaurus madagaskarensis*). Thus, the re-alignment of the data set in TNT was conducted using *Pamelaria dolichotrachela* as reference taxon because it is the earliest branching of the sampled allokotosaurs.

The hypotheses of incorporation of the centrale to the astragalus or complete loss of the former bone in archosauromorphs were tested comparing the length of the branch in which the centrale is absent as a different ossification (i.e. the Erythrosuchidae + Eucrocopoda clade) between the different topologies. In this way, the configuration that requires the highest number of steps in that branch will be the least parsimonious and its associated hypothesis will be rejected over the other. We used a scale in millimetres for the scaling of each morphogeometric configuration and, as a result, the differences in branch lengths and the total number of steps for each tree are expected to be relatively low (i.e. a magnitude of decimals). However, this optimization is based on maximum parsimony and any difference, regardless of how small it is (contrasting with probabilistic methods), is enough to prefer one hypothesis over others. We did not conduct a probabilistic Templeton test to explore statistical differences between optimizations because this analysis evaluates differences between alternative topologies, but not differences in character optimizations using the same topology.

## Supplementary information

Supplementary Information.

## Data Availability

All the data analysed in this study are available in the Supplementary Data Files.
