# Supplementary material for: New embryological and palaeontological evidence sheds light on the evolution of the archosauromorph ankle
Source: Sci Rep. 2020 Mar 20;10:5150. doi: 10.1038/s41598-020-62033-8 (PMC7083827; doi:10.1038/s41598-020-62033-8)
Supplement: Supplementary file 1 — Supplementary Information. [file 41598_2020_62033_MOESM1_ESM.pdf]

Supplementary information

New embryological and palaeontological evidence sheds light on the evolution of the archosauromorph ankle

M. Victoria Fernandez Blanco, Martín D. Ezcurra & Paula Bona

Contents:

i) – Institutional abbreviations.

ii) – Sampled embryonic specimens. Supplementary Table 1.

iii) – Sampled archosauromorph species. Supplementary Table 2.

iv) – Landmarks. Supplementary Figure 1 and Supplementary Table 3.

v) – Phylogenetic topologies and optimization of morphogeometric configurations. Supplementary Figures 2, 3.

vi) – Principal Component Analysis. Supplementary Figure 4.

vii) – Optimization of morphogeometric configurations. Supplementary Figures 5–16.

viii) – Data matrices in TNT format.

ix) – Coordinates for terminal taxa.

x) – References.

i) – Institutional abbreviations.

AMNH, American Museum of Natural History, New York, USA; **GR,** Ghost Ranch Ruth May Museum of Paleontology, New Mexico, USA; **ISIR,** Indian Statistical Institute, Reptiles, Kolkata, India; **MACN-Pv,** Museo Argentino de Ciencias Naturales "Bernardino Rivadavia", Paleovertebrados, Buenos Aires, Argentina; **MB,** Museum für Naturkunde Berlin, Germany; **MCP,** Museu de Ciências e Tecnologia da Pontifícia Universidade Católica do Rio Grande do Sul, Porto Alegre, Brazil; **MLP**, Museo de La Plata, Buenos Aires, Argentina; **NMQR,** National Museum, Bloemfontein, South Africa; **PVL,** Paleontología de Vertebrados, Instituto "Miguel Lillo", San Miguel de Tucumán, Argentina; **PVSJ,** División de Paleontología de Vertebrados del Museo de Ciencias Naturales y Universidad Nacional de San Juan, San Juan, Argentina; **QR,** Zimbabwe Natural History Museum, Bulawayo, Zimbabwe; **SMNS,** Staatliches Museum für Naturkunde Stuttgart, Stuttgart, Germany; **UCMP,** University of California Museum of Paleontology, Berkeley, USA; **UMZC,** University Museum of Zoology, Cambridge, UK; **USNM,** National Museum of Natural History (formerly United States National Museum), Smithsonian Institution, Washington, D.C., USA; **ZPAL,** Institute of Paleobiology of the Polish Academy of Sciences, Warsaw, Poland.

ii) – Sampled embryonic specimens.

Supplementary Table 1. Embryo specimens of *Caiman yacare* and *Caiman latirostris* used in our analyses.

C. yacare **MLP-R.6490**	***C. latirostris*** **MLP-R.6491**
CY-17/18-1	CL-20
CY-17/18-2	CL-21
CY-17/18-3	CL-22-1
CY-18-1	CL-22-2
CY-18-2	CL-22-3
CY-19	CL-22-4
CY-20	CL-22-5
CY-21-1	CL-22-6
CY-21-2	CL-22-7
CY-22-1	CL-22-8
CY-22-2	CL-22-9
CY-22-3	CL-22-10
CY-23-1	CL-22-11
CY-23-2	CL-23-1
CY-23-3	CL-23-2
CY-23-4	CL-23-3
CY-23-5	CL-23-4
CY-23-6	CL-23-5
CY-23-7	CL-23-6
CY-23-8	CL-23-7
CY-23-9	CL-23-8
CY-23-10	CL-23-9
CY-23-11	CL-23-10
CY-23-12	CL-23-11
CY-23-13	CL-24-1
CY-23-14	CL-24-2
CY-24-1	CL-25-1
CY-24-2	CL-25-2
CY-24-3	CL-25-3
CY-24-4	CL-25-4
CY-25-1	CL-25-5
CY-25-2	CL-25-6
CY-25-3	CL-27-28-1
CY-25-4	CL-27-28-2
	CL-27-28-3
	CL-27-28-4
	CL-27-28-5

iii) – Sampled archosauromorph species.

Supplementary Table 2. Sources used to generate the morphogeometric configurations that sample the astragalus and centrale of early archosauromorph species. Specimens studied at first-hand are indicated with an asterisk.

Species	Source of information
Adeopapposaurus mognai	PVSJ 610*
Aetosaurus ferratus	Schoch[1]: figure 12g*
Asilisaurus kongwe	Nesbitt et al.[2]: figure 51*
Azendohsaurus madagaskarensis	Nesbitt et al.[3]: figure 67a
Ceratosaurus nasicornis	USNM 4735*
Cryolophosaurus ellioti	Smith et al.[4]: figure 19c*
Dilophosaurus wetherilli	UCMP 37302*
Effigia okeeffeae	Nesbitt[5]: figure 49d
Eoraptor lunensis	Sereno et al.[6]: figure 88d*
Euparkeria capensis	UMZC T692*
Fasolasuchus tenax	PVL 3850*
Gracilisuchus stipanicicorum	PVL 4597*
Herrerasaurus ischigualastensis	PVSJ 373*
Jaklapallisaurus assymetrica	ISIR 274*
Lewisuchus admixtus	MACN-Pv 18954*
Liliensternus liliensterni	MB R 2175*
Mandasuchus tanyauchen	Butler et al.[7]: figure 25a*
Lagosuchus talampayensis (= *Marasuchus lilloensis*)	Sereno & Arcucci[8]: fig. 11a*
Megapnosaurus rhodesiensis	QR 691*
Nundasuchus songeaensis	Nesbitt et al.[9]: figure 11*
Parasuchus hislopi	ISIR 43*
Pisanosaurus mertii	PVL 2577*
Plateosaurus engelhardti	SMNS 13200*
Polesinesuchus aurelioi	Roberto-Da-Silva et al.[10]: figure 29[a]*
Postosuchus alisonae	Peyer et al.[11]: figure 9d
Postosuchus kirkpatricki	Weinbaum[12]; figure 22b
Powellvenator podocitus	PVL 4414*
Proterosuchus alexanderi	NMQR 1484*
Proterosuchus sp.	AMNH 2237*
Pseudopalatus sp.	Nesbitt[13]: 43e
Rauisuchus tiradentes	Lautenschlager & Rauhut[14]; figure 15a*
Rhadinosuchinae indet.	CRILAR-Pv 492*
Riojasuchus tenuisceps	PVL 3827*
Saturnalia tupiniquim	MCP 3844-PV*
Sctuellosaurus lawleri	UCMP 130580*
Shansisuchus shansisuchus	Gower[15]: figure 9a*
Shringasaurus indicus	ISIR 1059*
Silesaurus opolensis	ZPAL Ab III 361*
Smilosuchus gregorii	USNM 18313*
Tawa hallae	GR 242
Vancleavea campi	Nesbitt et al.[16]: figure 17d

iv) – Landmarks.

Supplementary Table 3. Description of landmarks and semilandmarks used in this study. Type II landmarks are mathematical points that refer to geometric structures and type III landmarks are points defined in relation to others. Semilandmarks are a particular type of type III landmarks[17]. The medial margin of the astragalus or astragalus+centrale is sampled by landmarks 2, 6 and 9 and semi-landmarks 1 and 2.

Landmark 1	Most lateral edge of the tibial facet	Type II
Landmark 2	Most medial edge of the tibial facet	Type II
Landmark 3	Most medial edge of the fibular facet	Type II
Landmark 4	Most lateral edge of the fibular facet	Type II
Landmark 5	Most ventral point of the astragalar body at the level of the half of its maximum width	Type II
Landmark 6	Midpoint between L1 and L2	Type III
Landmark 7	Midpoint between L3 and L4	Type III
Landmark 8	Ventral margin of the calcaneal facet	Type II
Landmark 9	Midpoint between L2 and L5	Type III
Semilandmark 1	Midpoint between L2 and L6	-
Semilandmark 2	Midpoint between L2 and L9	-

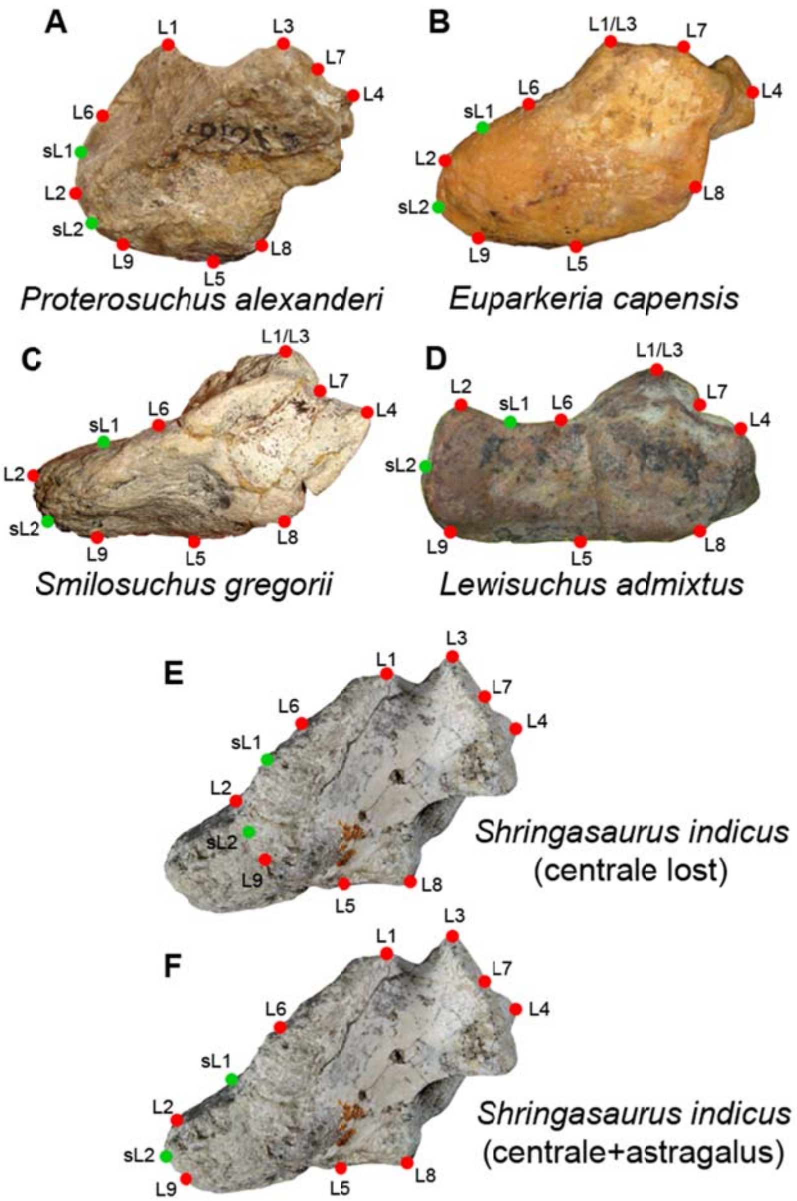

Supplementary Figure 1. Selected examples of morphogeometric configurations in (A) *Proterosuchus alexanderi* (NMQR 1484), (B) *Euparkeria capensis* (UMZC T692), (C) *Smilosuchus gregorii* (USNM 18313), (D) *Lewisuchus admixtus* (MACN-Pv 18954), and (E, F) *Shringasaurus indicus* (ISIR 1059). (A–E) Configurations that sample only the astragalus showing the complete loss of the centrale as an independent ossification. (F) Configuration that samples the astragalus and the centrale as a single unit to test the hypothesis that the former element was incorporated to the astragalus to form a single ossification centre. Astragali and cantrale not to scale.

v) – Phylogenetic topologies and optimization of morphogeometric configurations.

The morphogeometric configurations were optimized using two informal supertrees in TNT 1.5[18]. The first supertree is mainly based on the phylogenetic relationships of early archosauromorphs found by Ezcurra[19] and subsequent iterations of this analysis[20,21] (Supplementary Figure 2). In this supertree, the phylogenetic relationships among paracrocodylomorph pseudosuchians are based on Nesbitt & Desojo[22] and Butler et al.[7]. The second supertree is based on the phylogenetic trees recovered by Nesbitt[13]and subsequent iterations of this analysis[7,22] (Supplementary Figure 3). In this supertree, the phylogenetic position of *Shringasaurus indicus* is based on Sengupta et al.[21] and the erythrosuchid *Erythrosuchus africanus* was interchanged by *Shansisuchus shansisuchus* (following the phylogenetic position recovered by Ezcurra[19] for the latter species). In addition, in both supertrees, the phylogenetic relationships among non-sauropodomorph dinosaurs are based on Ezcurra[23] and within Sauropodomorpha are based on Müller et al.[24], and the Rhadinosuchinae indet. was considered as a sample of proterochampsids. As a result, both supertrees differ in the placement of *Euparkeria capensis*, *Vancleavea campi*, phytosaurs, the Rhadinosuchinae indet, *Nundasuchus songeaensis* and *Riojasuchus tenuisceps*.

The sampled morphogeometric configurations were optimized in the two supertrees (see Goloboff & Catalano[18]) in order to quantify the numbers of transformations recovered in the branch where the centrale is lost as a separate ossification (i.e. the branch leading to Erythrosuchidae+Eucrocopoda). The optimized branch lengths were compared between both morphogemetric configurations in order to determine which one is more parsimonious. The configuration that requires the lower number of steps for the branch where the centrale is lost will be considered as the preferred hypothesis.

The branch lengths were calculated for all the landmarks (Supplementary Figures 5, 6, 9, 10) and for the landmarks that specifically sample the medial margin of the proximal tarsus (see Landmarks section) (Supplementary Figures 7, 8, 11, 12) using the commands "lmark wts = 0 0/.; lmark wts = 1 0/5 9 1 10 8;" in TNT 1.5. In both cases, the results were congruent and favoured the

incorporation of the centrale to the astragalus over the complete loss of the element. The optimization of the morphogeometric configurations are also shown graphically (command “lmark”; Supplementary Figures 13–16) and the landmarks were re-ordered with the following command line “lmark connect 0 2-6 6-3 3-7 7-4 4-8 8-10 10-1 1-9 9-5 5-0 0-2;” in TNT 1.5.

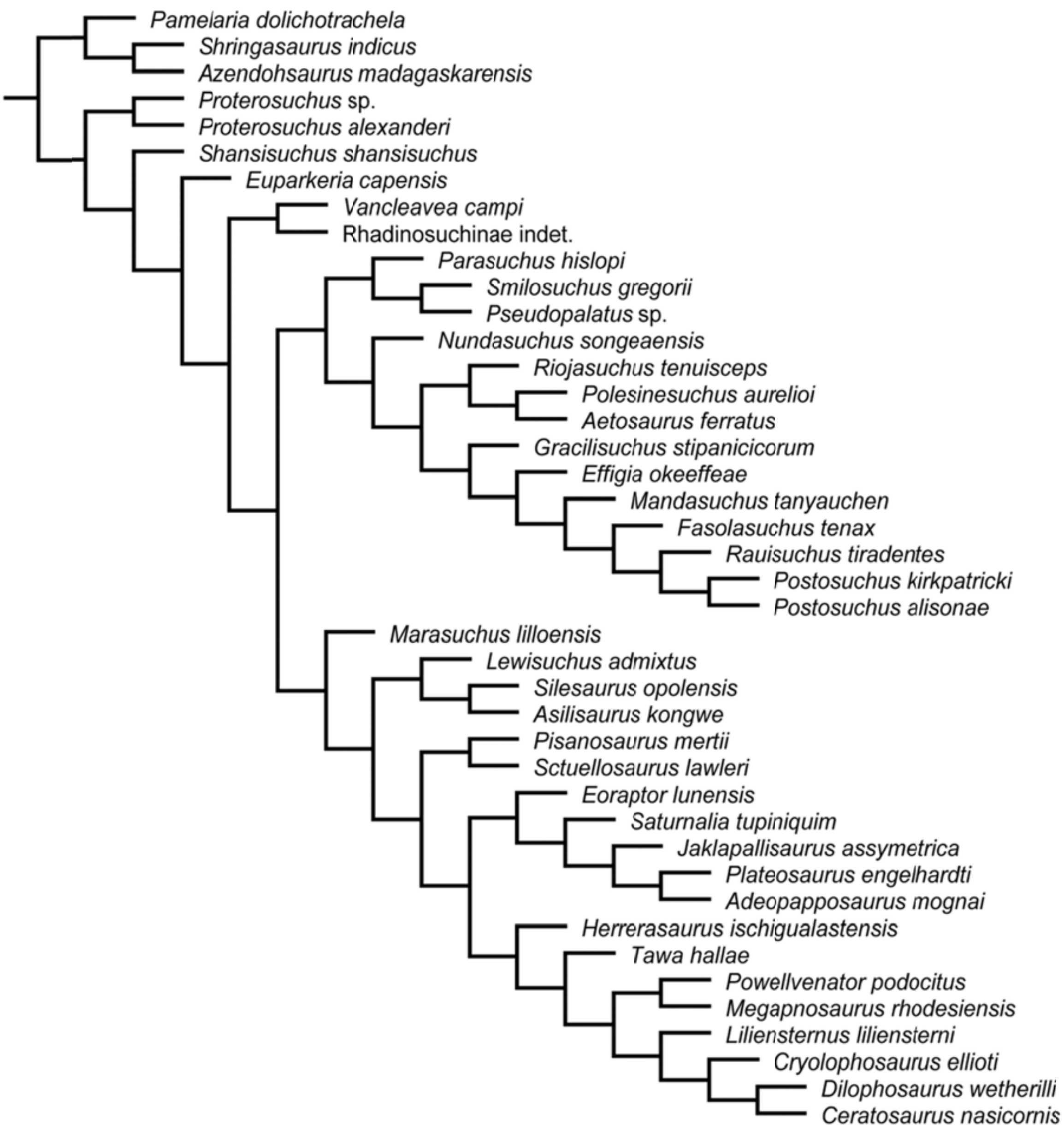

Supplementary Figure 2. Informal supertree mainly based on the phylogenetic analysis of Ezcurra[19] and its subsequent modifications.

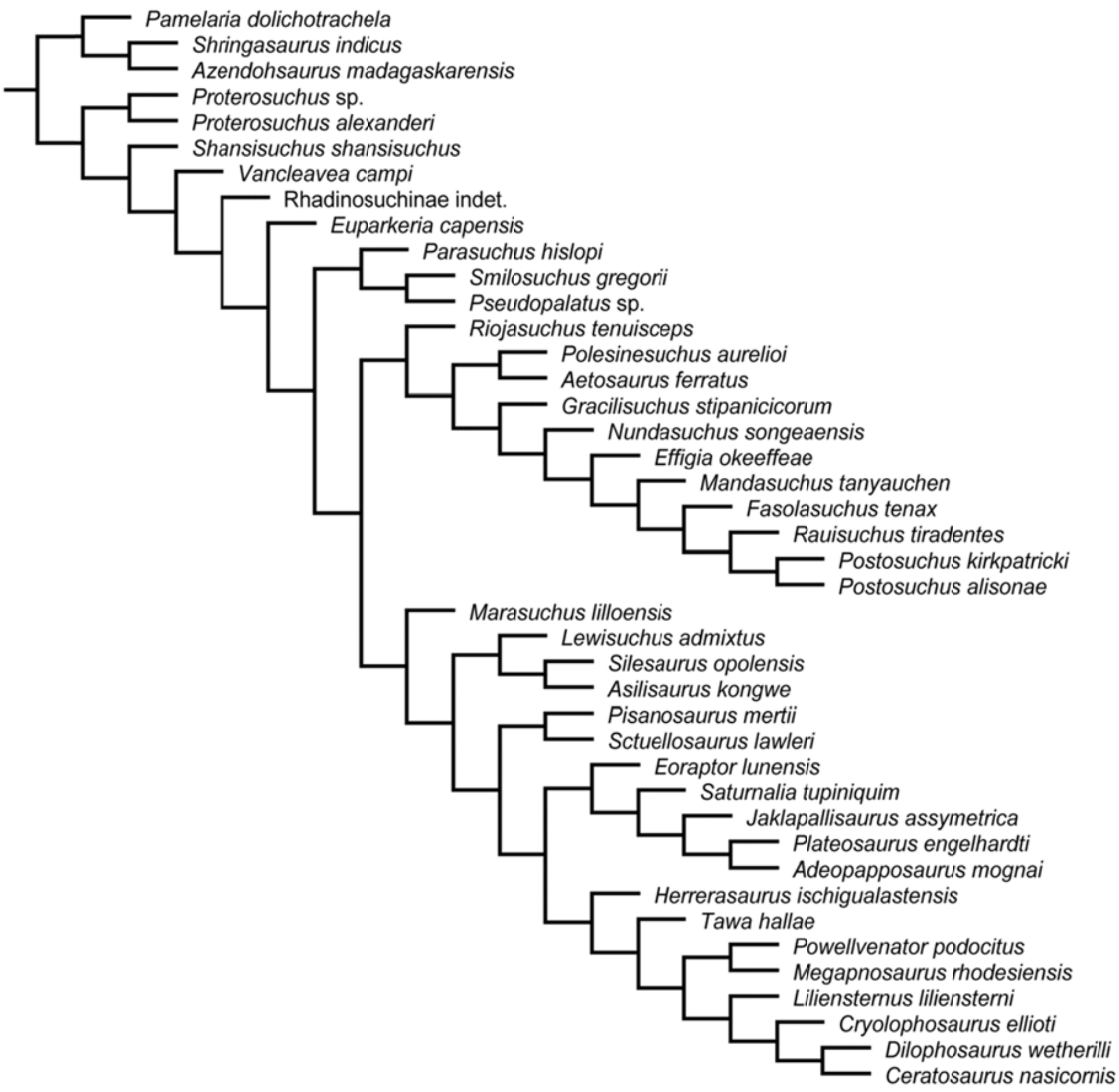

Supplementary Figure 3. Informal supertree mainly based on the phylogenetic analysis of Nesbitt[13] and its subsequent modifications.

vi) – Principal Component Analysis.

The morphogeometric configurations of all the terminals (including terminals sampled twice, i.e. astragalus only versus astragalus+centrale) were included in a Principal Component Analysis in order to explore their distribution in the morphospace. This analysis was conducted because it is expected that the terminals sampled under the different hypotheses were clustered in different regions of the morphospace. The terminals sampled with the more plausible hypothesis would be placed closer to the centroid of the terminals that lack a centrale as an independent ossification. As a result, the Sum of Variances (SoV) of the terminals with the most plausible hypothesis should be lower. The SoV was calculated in R for both terminal samplings.

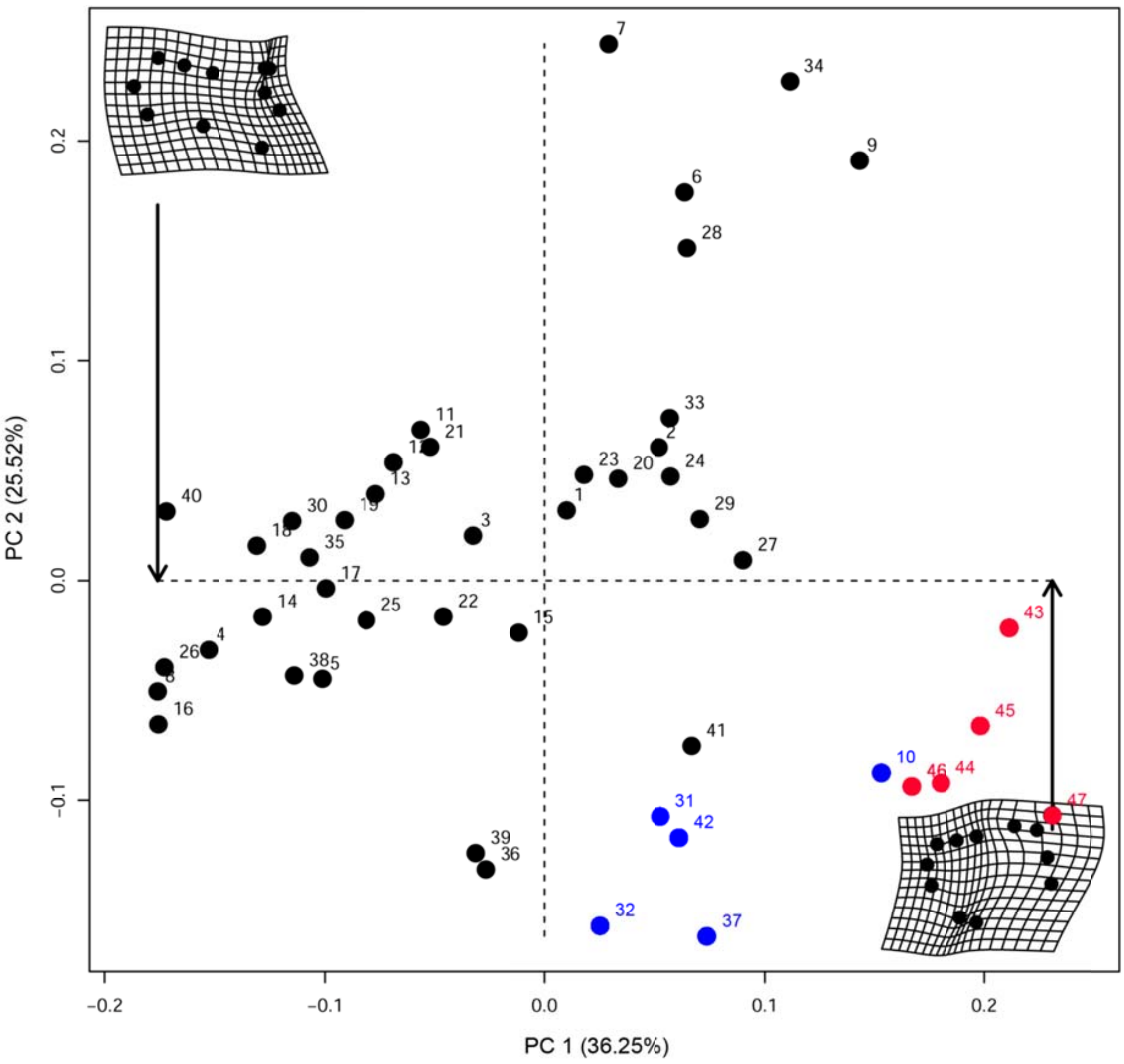

Supplementary Figure 4. Biplot of the first two components recovered from the Principal Component Analysis. 1–9, 11–30, 33–36, 38–41, taxa lacking a centrale as a separate ossification; 10, 31, 32, 37, 42–47, taxa with a centrale as a separate ossification. Blue dots indicate terminals with a morphogeometric configuration sampling the astragalus+centrale and red dots are the same terminals as the blue ones but the morphogeometric configurations do not sample the centrale.

vii) – Optimization of morphogeometric configurations.

Supplementary Figure 5. Optimization of the morphogeometric configuration sampling only the astragalus in the phylogenetic topology of Ezcurra[19] and its subsequent modifications. Branch lengths indicate transformations including all landmarks.

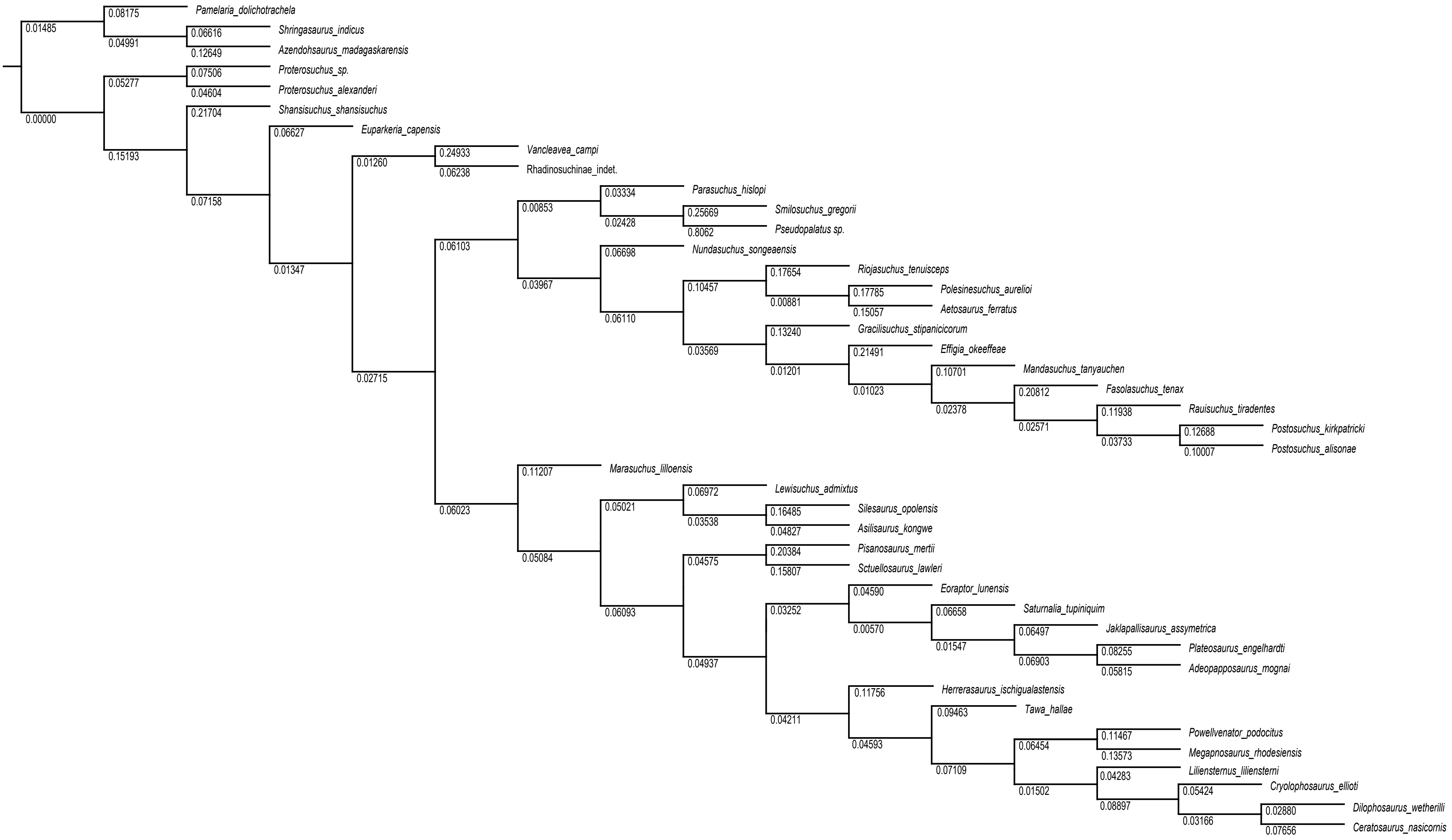

Supplementary Figure 6. Optimization of the morphogeometric configuration sampling the astragalus+centrale in the phylogenetic topology of Ezcurra[19] and its subsequent modifications. Branch lengths indicate transformations including all landmarks.

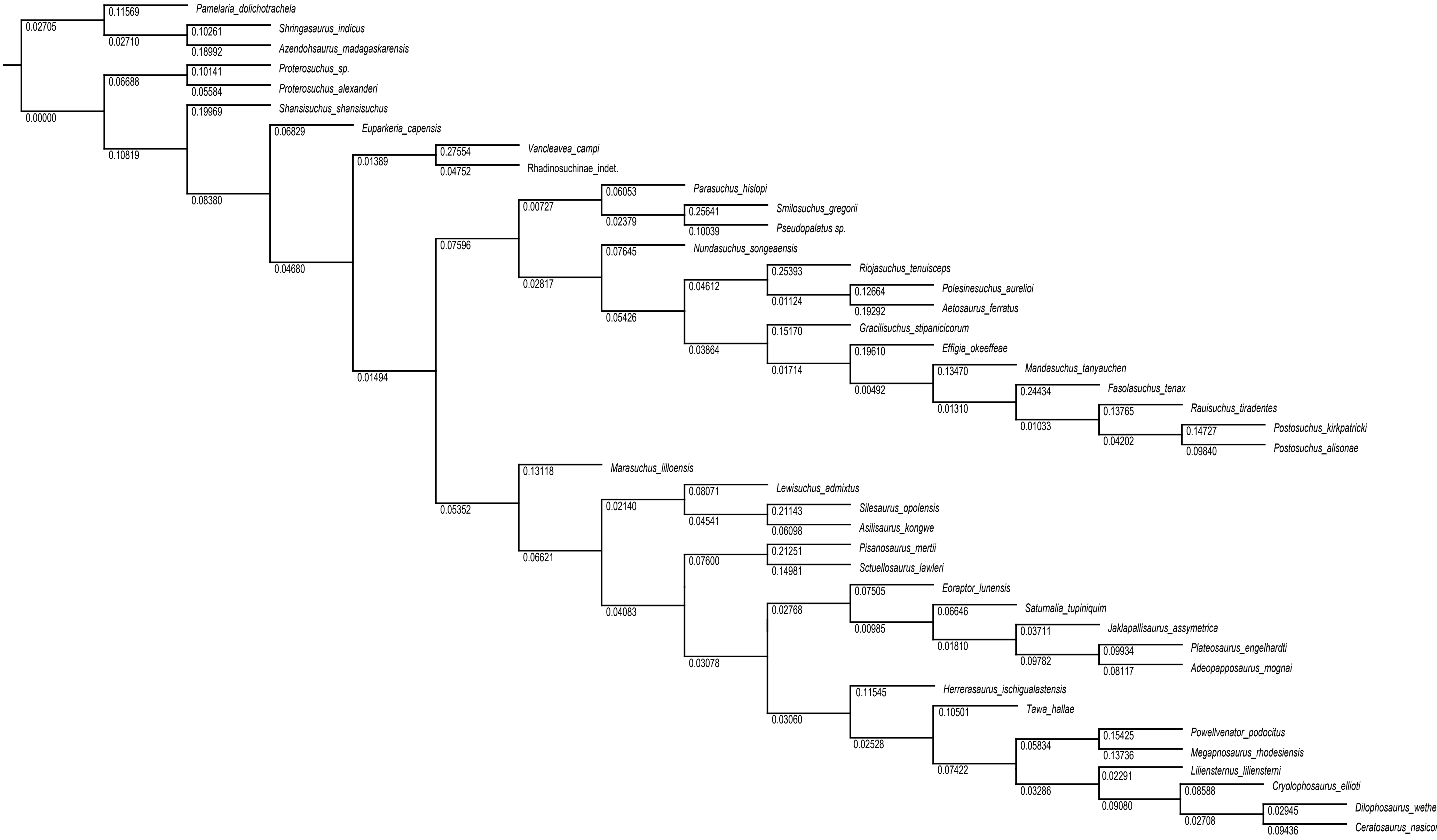

Supplementary Figure 7. Optimization of the morphogeometric configuration sampling only the astragalus in the phylogenetic topology of Ezcurra[19] and its subsequent modifications. Branch lengths indicate transformations of only the landmarks that sample the medial margin of the proximal tarsus (landmarks 2, 6 and 9 and semi-landmarks 1 and 2).

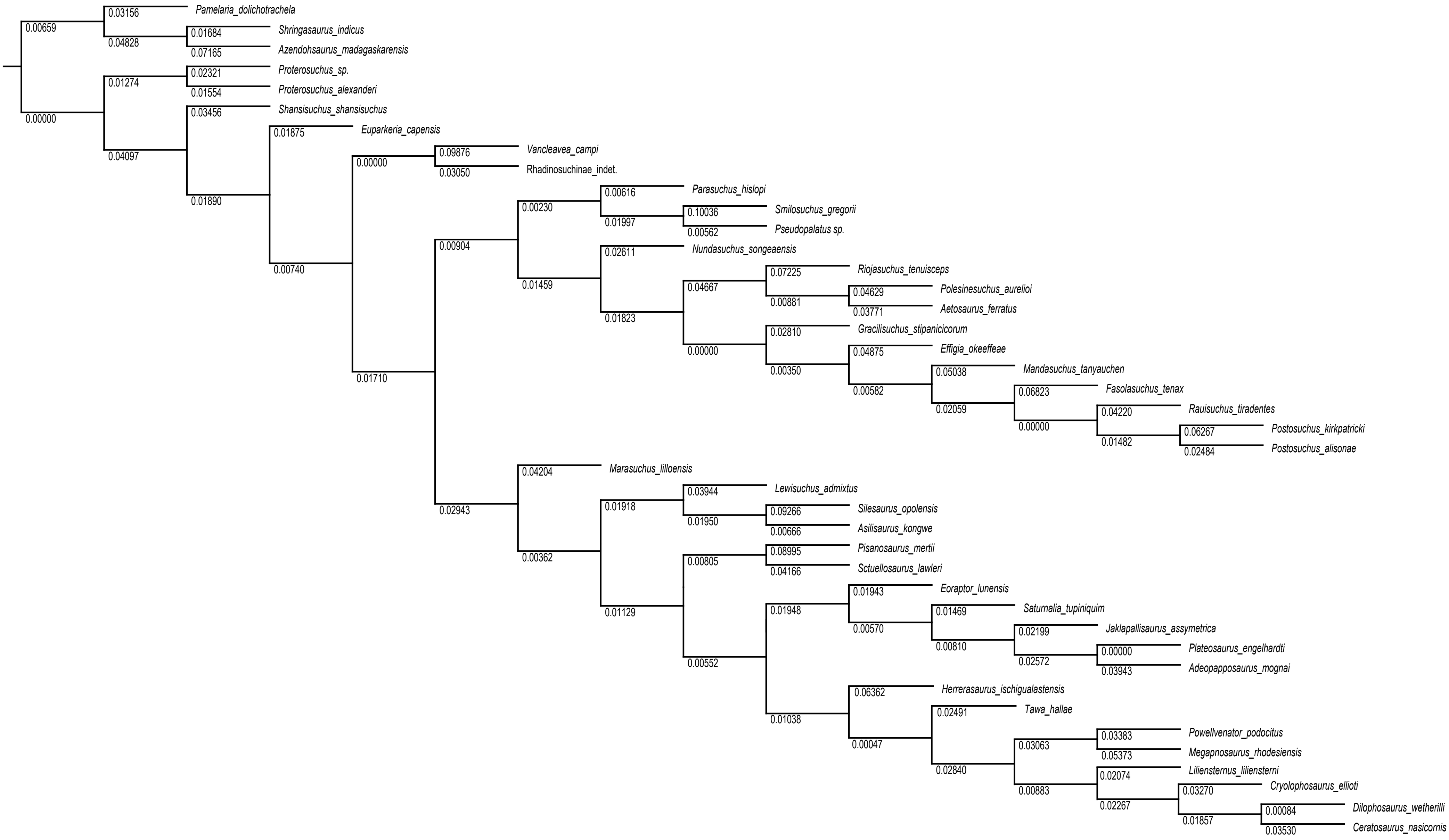

Supplementary Figure 8. Optimization of the morphogeometric configuration sampling the astragalus+centrale in the phylogenetic topology of Ezcurra[19] and its subsequent modifications. Branch lengths indicate transformations of only the landmarks that sample the medial margin of the proximal tarsus (landmarks 2, 6 and 9 and semi-landmarks 1 and 2).

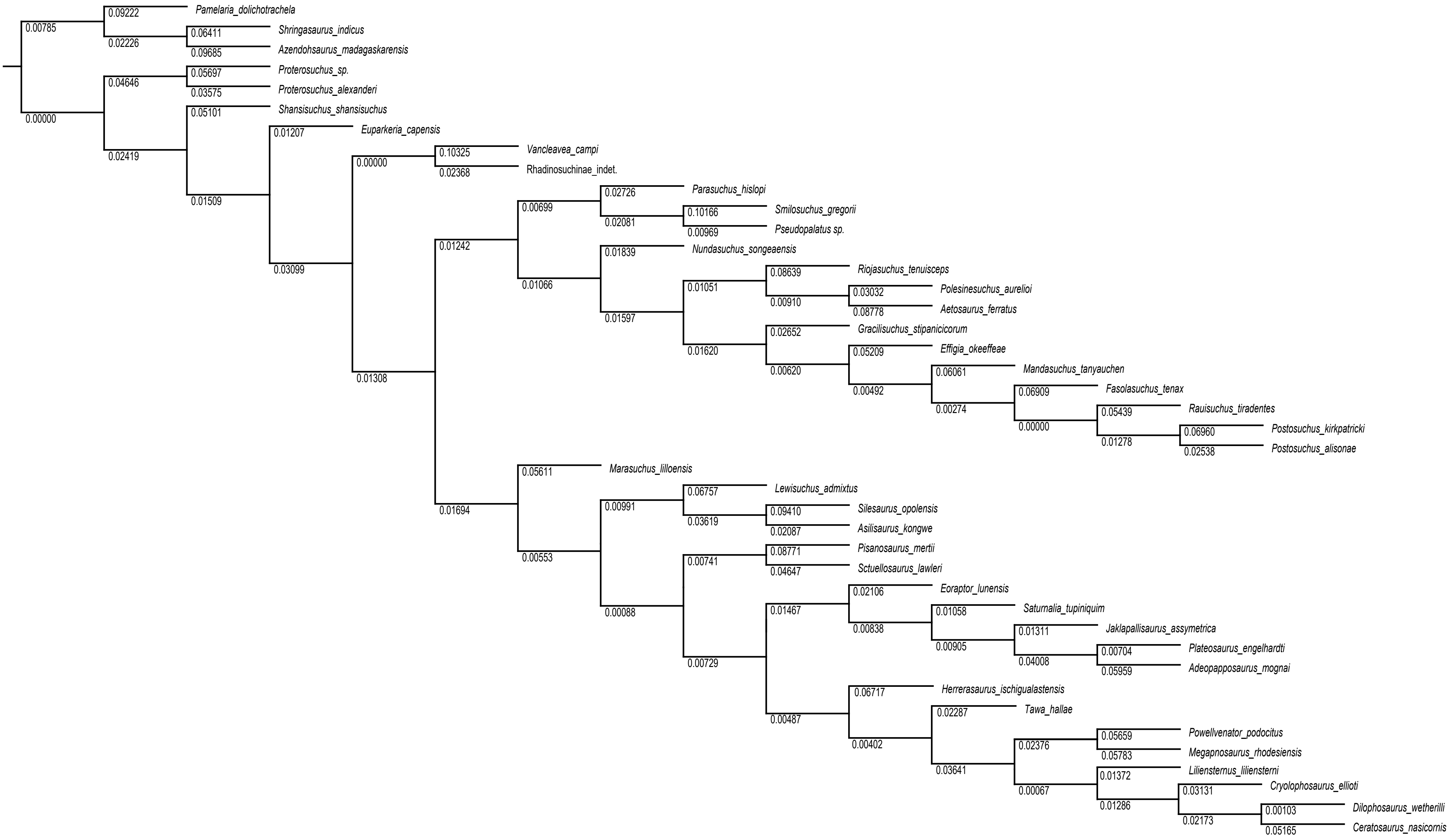

Supplementary Figure 9. Optimization of the morphogeometric configuration sampling only the astragalus in the phylogenetic topology of Nesbitt[13] and its subsequent modifications. Branch lengths indicate transformations including all landmarks.

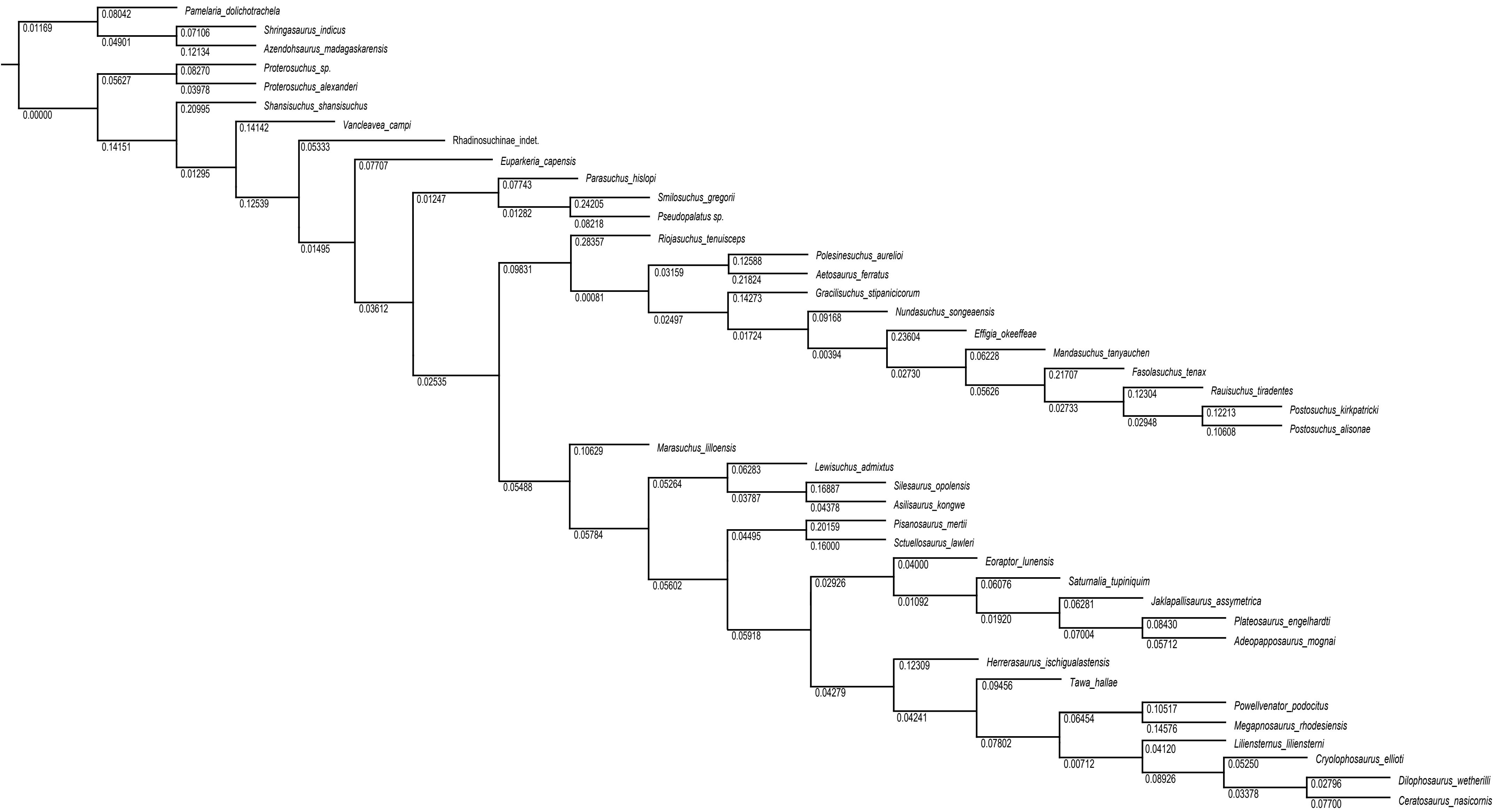

Supplementary Figure 10. Optimization of the morphogeometric configuration sampling the astragalus+centrale in the phylogenetic topology of Nesbitt[13] and its subsequent modifications. Branch lengths indicate transformations including all landmarks.

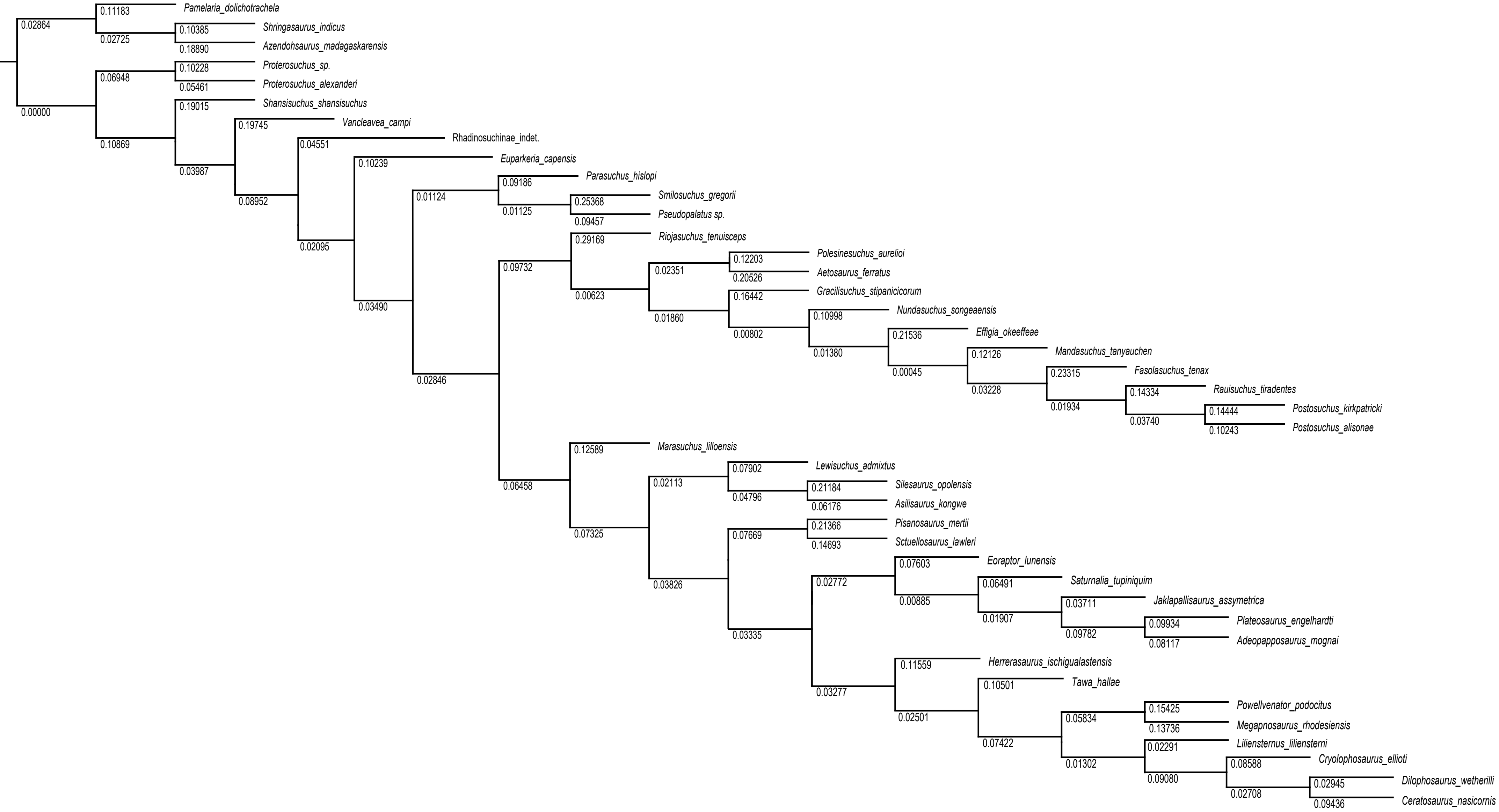

Supplementary Figure 11. Optimization of the morphogeometric configuration sampling only the astragalus in the phylogenetic topology of Nesbitt[13] and its subsequent modifications. Branch lengths indicate transformations of only the landmarks that sample the medial margin of the proximal tarsus (landmarks 2, 6 and 9 and semi-landmarks 1 and 2).

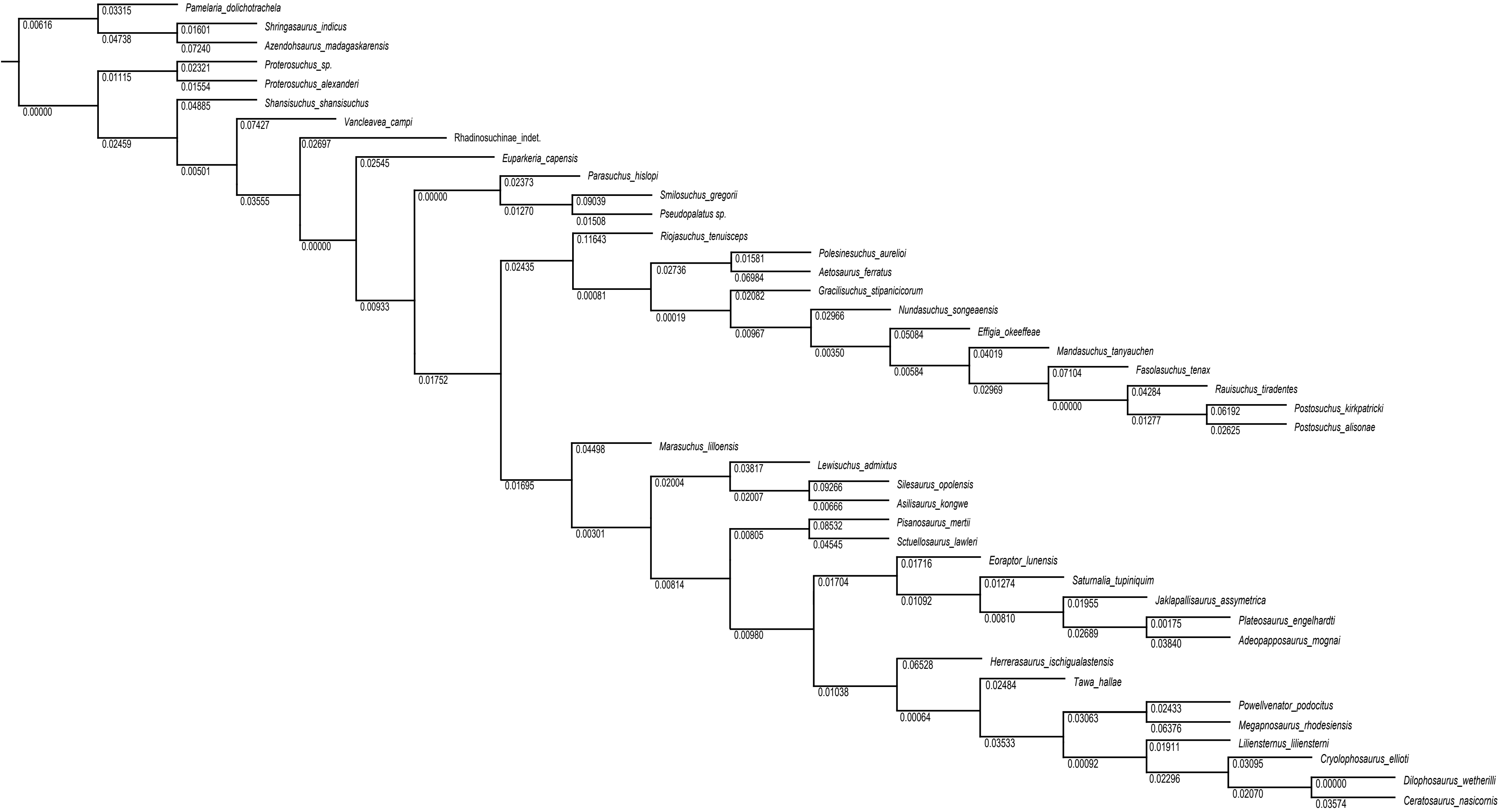

Supplementary Figure 12. Optimization of the morphogeometric configuration sampling the astragalus+centrale in the phylogenetic topology of Nesbitt[13] and its subsequent modifications. Branch lengths indicate transformations of only the landmarks that sample the medial margin of the proximal tarsus (landmarks 2, 6 and 9 and semi-landmarks 1 and 2).

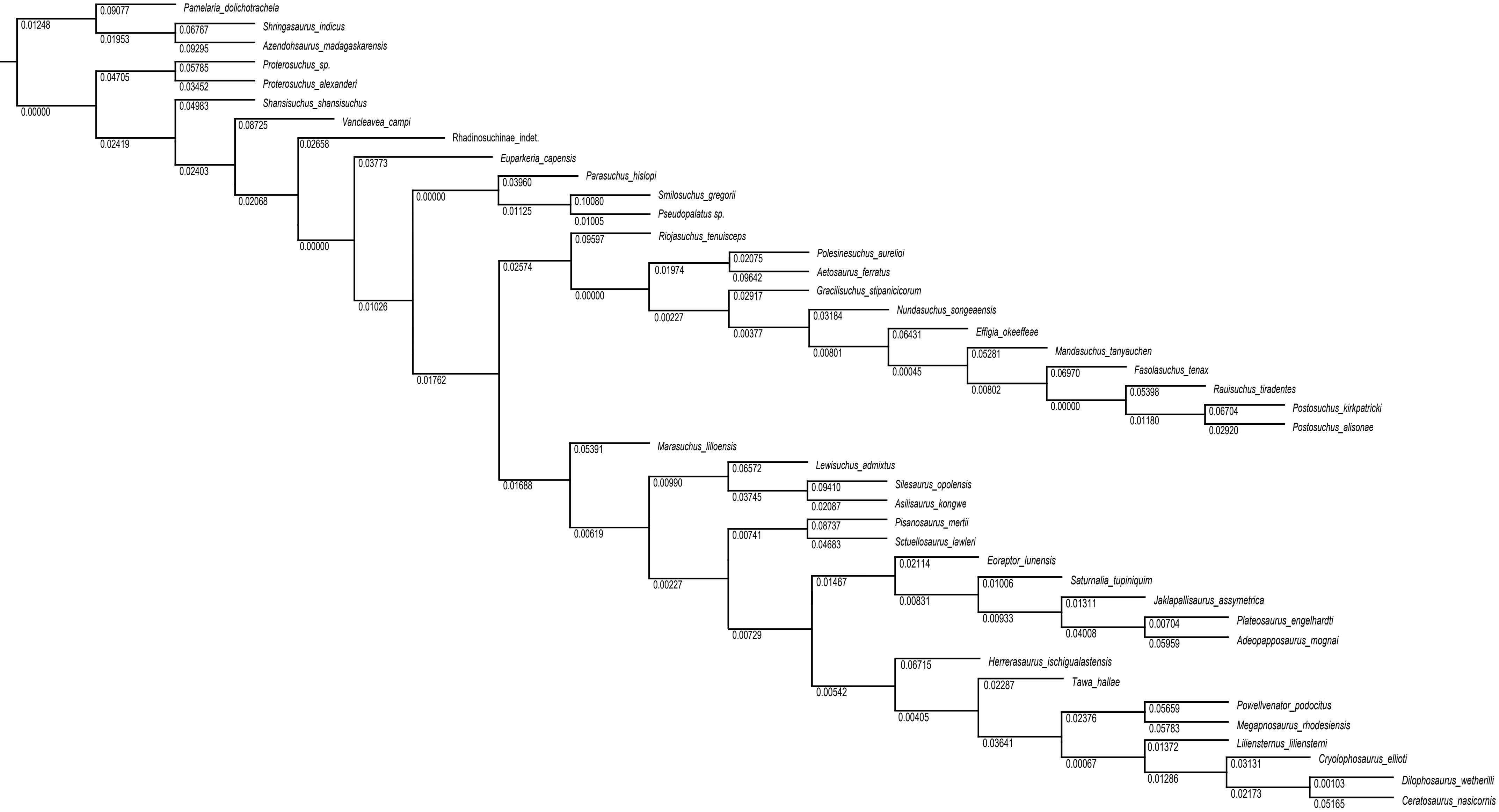

Supplementary Figure 13. Morphogeometric character (astragalus only) - Ezcurra phylogeny: 11 points (score: 6.37848)

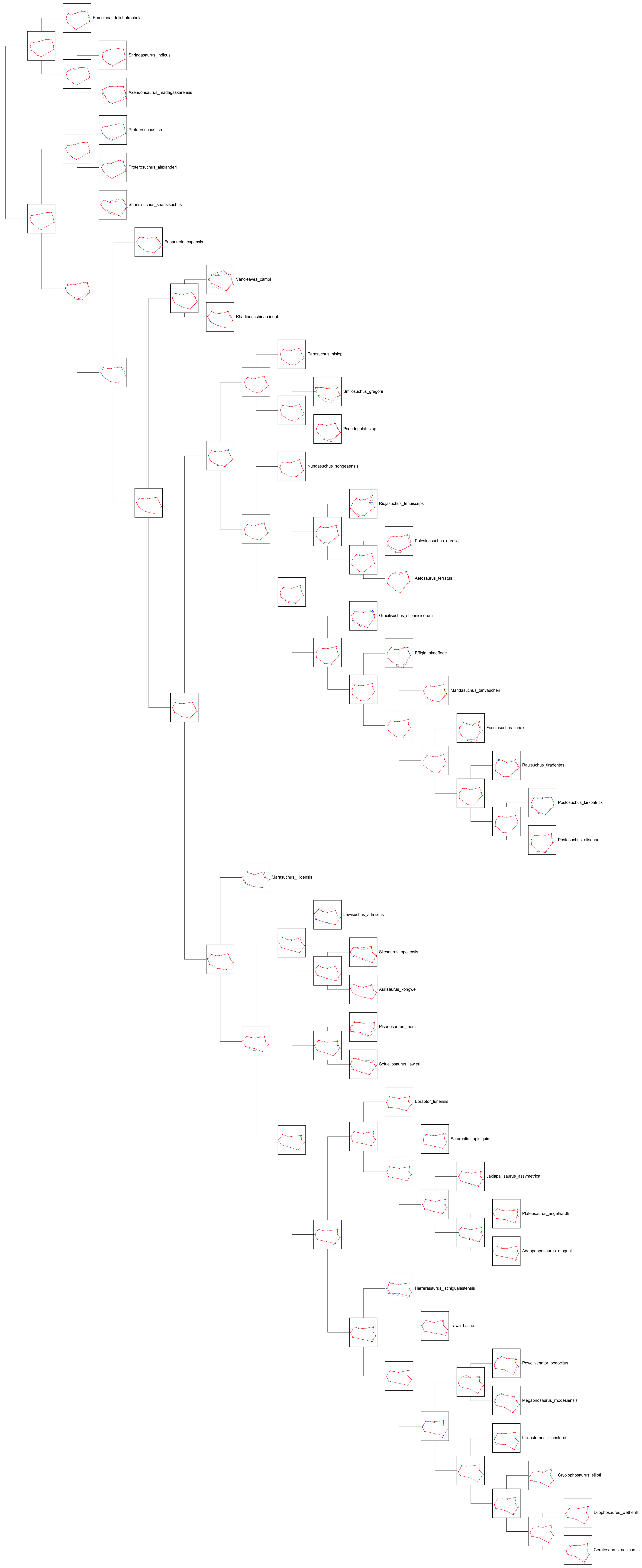

Supplementary Figure 14. Morphogeometric character (astragalus+centrale) - Ezcurra phylogeny: 11 points (score: 6.87994)

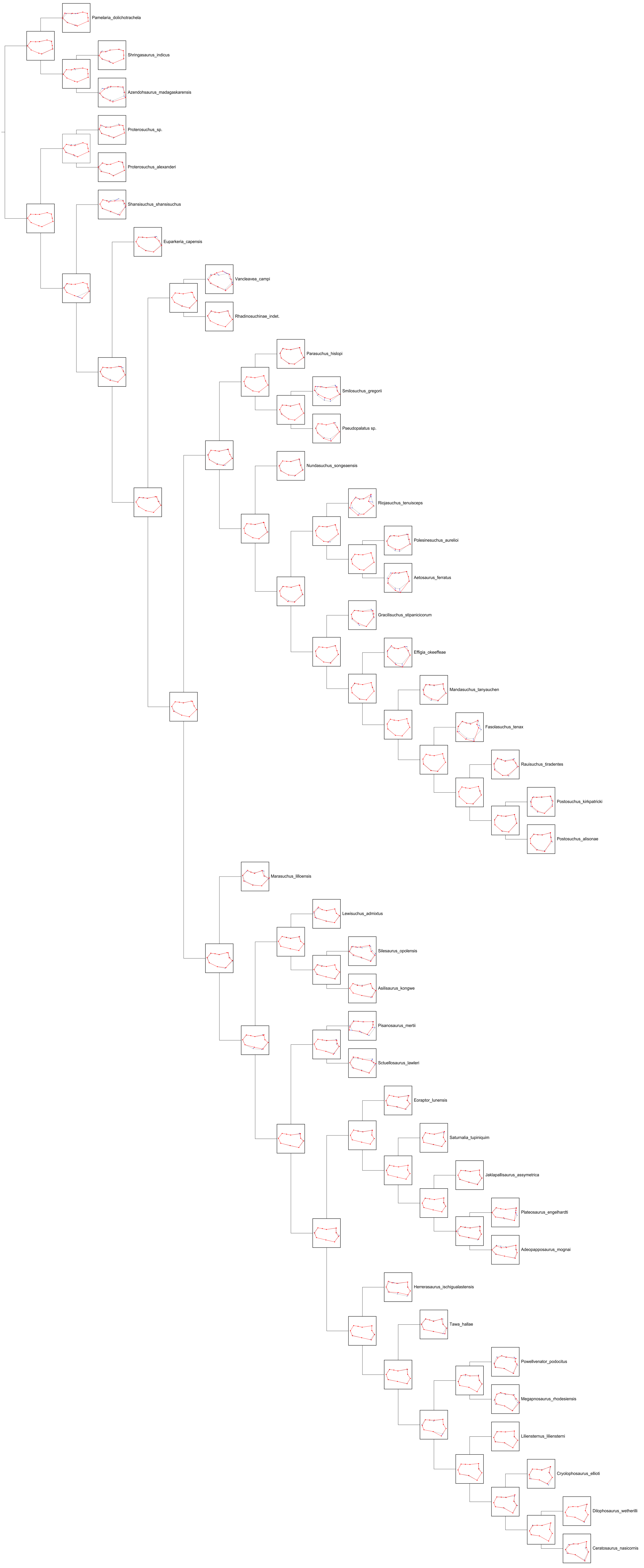

Supplementary Figure 15. Morphogeometric character (astragalus only) - Nesbitt phylogeny: 11 points (score: 6.40126)

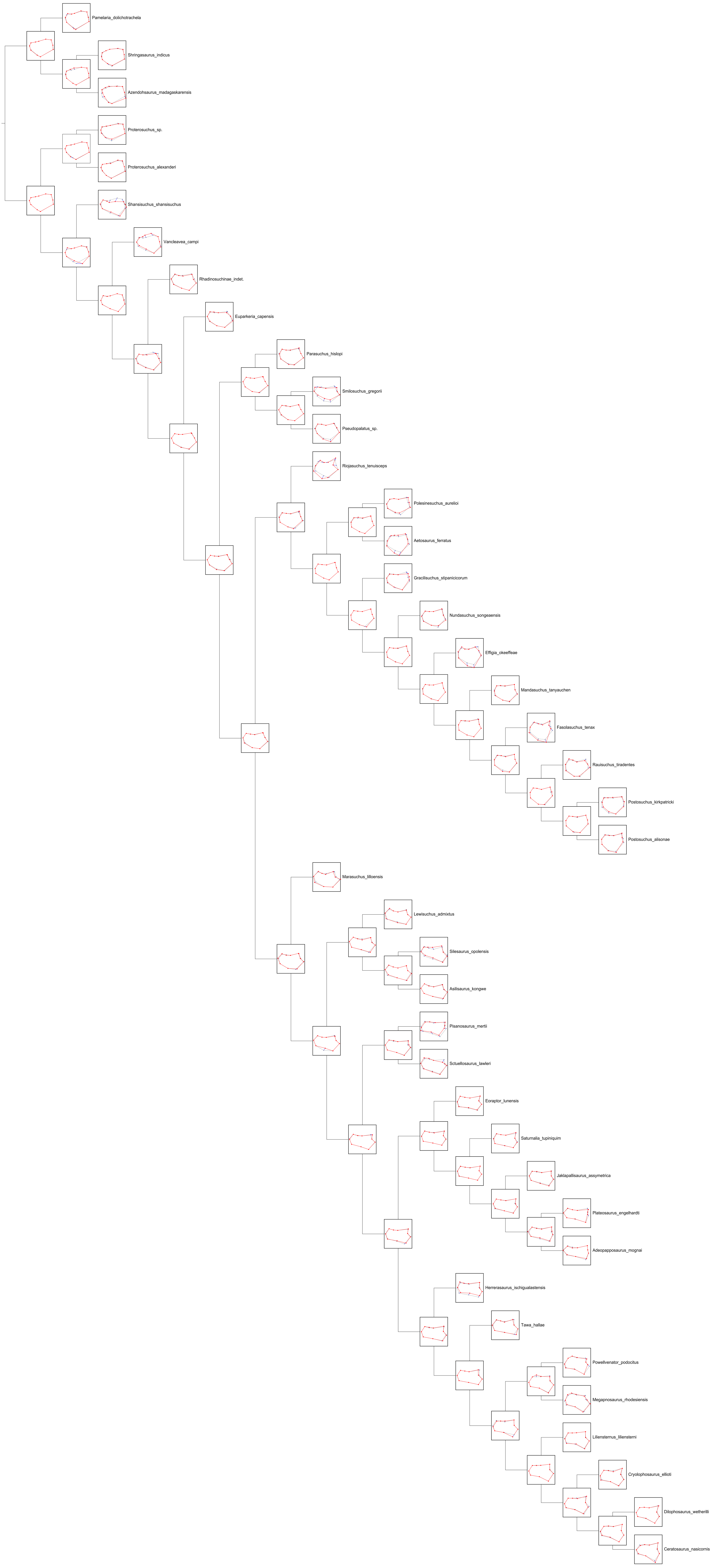

Supplementary Figure 16. Morphogeometric character (astragalus+centrale) - Nesbitt phylogeny: 11 points (score: 6.91867)

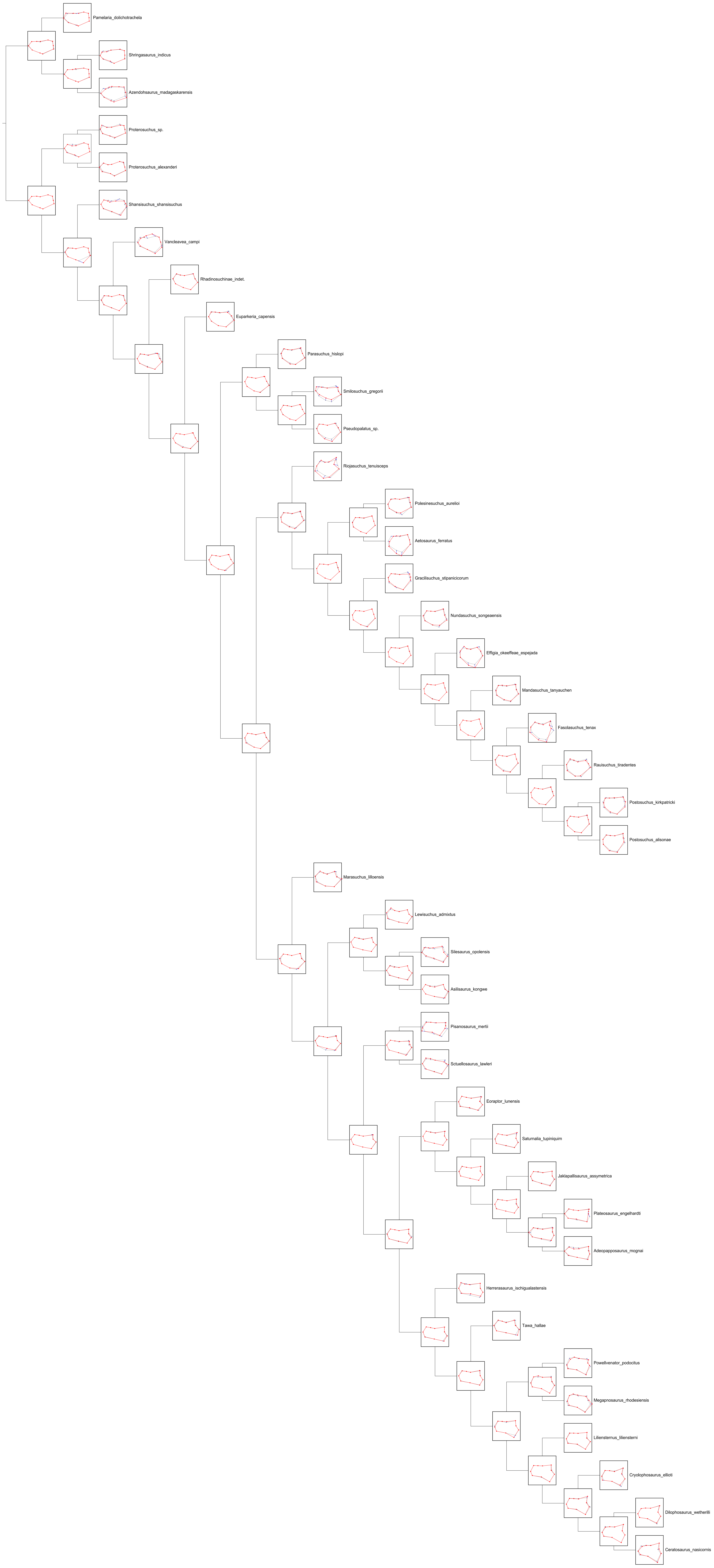

viii) – Phylogenetic matrices in TNT format.

Phylogenetic matrix of the centrale+astragalus hypothesis in Ezcurra (2016) topology

xread

'Landmark data, imported from TPS to TNT' 1 42

&[landmark 2d]

Gracilisuchus_stipanicicorum_PV 0.280821,0.145902 -0.285444,0.123894 0.280533,0.145049 0.317436,-0.032226 -0.144156,-0.245331 -0.023047,0.114079 0.311828,0.060858 0.109562,-0.325880 -0.335212,-0.113146 -0.166368,0.122012 -0.368342,0.005558

Pseudopalatus_UCMP_27235_espeja 0.211945,0.172573 -0.276128,0.158861 0.212150,0.172528 0.379872,-0.094089 -0.119957,-0.276830 -0.027447,0.119186 0.304839,0.046001 0.111602,-0.359192 -0.294566,-0.115293 -0.156408,0.150214 -0.349974,0.027174

Rhadinosuchinae_espejada 0.235592,0.160439 -0.278485,0.170515 0.235539,0.160451 0.364209,-0.096346 -0.084149,-0.239420 -0.039822,0.109879 0.282239,0.009307 0.145566,-0.306042 -0.319216,-0.126833 -0.164667,0.130178 -0.395435,0.029614

Asilisaurus_kongwe_1 0.231766,0.127483 -0.310086,0.176288 0.232105,0.127409 0.372916,-0.090689 -0.074179,-0.197355 -0.035012,0.077561 0.278289,-0.020235 0.241960,-0.270923 -0.334919,-0.097730 -0.179732,0.112767 -0.424789,0.051072

Sctuellosaurus_lawleri_UCMP_130 0.238992,0.069362 -0.319598,0.185766 0.239329,0.069289 0.371529,-0.013816 -0.064540,-0.216065 -0.036820,0.121771 0.309502,0.017282 0.166444,-0.285144 -0.317827,-0.135881 -0.178268,0.173294 -0.422423,0.018328

Effigia_okeeffeae_espejada 0.181292,0.180946 -0.222711,0.199904 0.182057,0.180779 0.353166,-0.065158 -0.078814,-0.352378 -0.016806,0.121325 0.289215,0.071344 0.123602,-0.418169 -0.337081,-0.118386 -0.132987,0.132965 -0.338961,0.064572

Fasolasuchus_tenax_PVL_3850 0.253114,0.202693 -0.217987,0.177191 0.253711,0.202127 0.279551,-0.018580 -0.119446,-0.347555 0.033602,0.091643 0.227199,0.080210 0.128585,-0.415215 -0.379719,-0.123201 -0.097271,0.127710 -0.349651,0.029666

Adeopapposaurus_mognai_PVSJ_610 0.282068,0.128194 -0.316618,0.142764 0.282860,0.127541 0.306283,-0.089810 -0.087015,-0.167710 -0.015251,0.079255 0.286593,0.001246 0.239009,-0.248352 -0.341142,-0.099530 -0.172336,0.082893 -0.461887,0.033896

Aetosaurus_ferratus 0.221016,0.212191 -0.212754,0.152141 0.221797,0.212021 0.306866,-0.076872 -0.107038,-0.349739 0.002010,0.169220 0.288269,0.061648 0.044622,-0.414676 -0.343177,-0.149268 -0.113047,0.167232 -0.323365,-0.007945

Azendohsaurus_madagaskarensis 0.155484,0.177603 -0.253514,0.099892 0.313627,0.170727 0.398476,-0.139323 -0.077993,-0.240054 -0.061205,0.183948 0.364266,0.014158 0.010045,-0.270762 -0.293749,-0.138597 -0.142012,0.162252 -0.393935,-0.029348

Ceratosaurus_nasicornis_USNM_47 0.260384,0.188426 -0.270859,0.117473 0.260571,0.188385 0.304046,-0.118458 -0.109695,-0.182088 -0.002623,0.131361

0.242874,0.016225 0.176105,-0.379435 -0.332696,-0.119656 -0.139719,0.142637 -0.397752,-0.012797

Cryolophosaurus_ellioti 0.275981,0.187556 -0.280762,0.141179 0.276061,0.187539 0.319617,-0.137460 -0.082524,-0.189895 0.003046,0.098426 0.225654,0.016171 0.154799,-0.311508 -0.374933,-0.137823 -0.142281,0.129222 -0.390423,-0.018736

Dilophosaurus_wetherilli_UCMP_3 0.285762,0.185923 -0.290150,0.127015 0.285954,0.185881 0.305374,-0.126803 -0.099699,-0.177596 0.000354,0.124000 0.227881,0.002331 0.170343,-0.319486 -0.347134,-0.131795 -0.149347,0.137927 -0.395391,-0.020690

Eoraptor_lunensis_espejada 0.292140,0.138129 -0.311161,0.159598 0.292255,0.138104 0.326955,-0.096790 -0.076981,-0.178565 -0.019271,0.098043 0.240865,0.000547 0.181875,-0.254873 -0.364989,-0.142673 -0.173346,0.123883 -0.409339,-0.001970

Euparkeria_capensis_UMZC_T692 0.207978,0.132115 -0.298494,0.159237 0.207528,0.130824 0.399862,-0.110467 -0.093993,-0.246380 -0.045208,0.131030 0.324902,0.027230 0.147825,-0.295200 -0.297603,-0.110638 -0.176826,0.152184 -0.382149,0.027470

Herrerasaurus_ischigualastensis 0.266417,0.129006 -0.326755,0.184869 0.266941,0.129329 0.332181,-0.138401 -0.047648,-0.162082 -0.066896,0.114386 0.259654,-0.027259 0.247691,-0.251830 -0.326746,-0.128018 -0.210728,0.137688 -0.404895,0.023433

Jaklapallisaurus_assymetrica_IS 0.263404,0.143692 -0.310070,0.149254 0.262899,0.142569 0.334747,-0.095232 -0.075158,-0.207747 -0.020002,0.108611 0.265164,0.005028 0.187052,-0.281820 -0.344630,-0.111940 -0.169683,0.141082 -0.402275,0.013961

Lewisuchus_admixtus_MACN_Pv 0.225396,0.128774 -0.279277,0.189770 0.225322,0.129215 0.367206,-0.072601 -0.044945,-0.225344 -0.029521,0.083074 0.270934,-0.000356 0.212246,-0.276670 -0.385253,-0.110285 -0.165831,0.115305 -0.415933,0.051264

Liliensternus_liliensterni_MB_R 0.246947,0.157768 -0.282966,0.158476 0.247151,0.158186 0.340692,-0.119370 -0.064973,-0.178199 -0.028103,0.144830 0.236666,-0.010774 0.183283,-0.334230 -0.350060,-0.141323 -0.160164,0.152369 -0.393323,-0.008515

Mandasuchus_tanyauchen 0.258585,0.176211 -0.281947,0.146094 0.258605,0.176206 0.344226,-0.105186 -0.103224,-0.268712 -0.028627,0.140169 0.273291,0.021536 0.097020,-0.311282 -0.320606,-0.138881 -0.164599,0.156176 -0.351389,-0.004279

Marasuchus_lilloensis 0.205578,0.156204 -0.239717,0.184354 0.205839,0.156147 0.418779,-0.074740 -0.101504,-0.260843 -0.028549,0.085696 0.262086,-0.007044 0.175031,-0.292011 -0.368528,-0.128384 -0.137333,0.125968 -0.394377,0.042652

Megapnosaurus_rhodesiensis_QR_6 0.205991,0.118544 -0.298372,0.153536 0.206233,0.118491 0.391376,-0.107030 -0.068261,-0.196795 -0.047089,0.167101 0.295280,0.004524 0.168059,-0.344895 -0.316549,-0.126779 -0.182273,0.201163 -0.366632,-0.008722

Nundasuchus_songeaensis 0.242588,0.195397 -0.266947,0.151488 0.242666,0.195380 0.344533,-0.137277 -0.098459,-0.266354 -0.014772,0.128739 0.280280,0.008691 0.115034,-0.284957 -0.326509,-0.140083 -0.144202,0.136261 -0.383496,0.008409

Parasuchus_hislopi_ISI_R_espeja 0.238135,0.185307 -0.275797,0.155702 0.237848,0.185416 0.366060,-0.110097 -0.082183,-0.290957 -0.027632,0.128997 0.291393,0.024366 0.079092,-0.303183 -0.323458,-0.126364 -0.159929,0.131396 -0.356432,0.020584

Pisanosaurus_mertii_PVL_2577 0.306788,0.121378 -0.295686,0.111931 0.305898,0.121718 0.287315,-0.053787 -0.106444,-0.171107 0.007471,0.107979 0.305802,0.031662 0.131667,-0.257955 -0.418414,-0.139000 -0.155686,0.121103 -0.406929,-0.050219

Plateosaurus_engelhardti_SMNS_1 0.309466,0.101402 -0.314687,0.149381 0.308939,0.101508 0.280336,-0.035319 -0.102096,-0.161476 -0.022030,0.084893 0.289476,0.029693 0.220628,-0.265438 -0.350255,-0.123033 -0.176607,0.109730 -0.445682,0.014408

Polesinesuchus_aurelioi 0.275458,0.184553 -0.281352,0.119149 0.275725,0.184495 0.351866,-0.101646 -0.103901,-0.254095 -0.009312,0.125547 0.313450,0.037664 0.019213,-0.274519 -0.328675,-0.144618 -0.152872,0.142895 -0.368649,-0.024660

Postosuchus_alisonae_espejada 0.264814,0.196466 -0.244884,0.142493 0.265231,0.196376 0.294899,-0.054599 -0.151351,-0.320471 0.013712,0.125314 0.269635,0.066305 0.093673,-0.360538 -0.338961,-0.134362 -0.122648,0.125689 -0.354142,0.011251

Postosuchus_kirkpatricki 0.255684,0.179216 -0.295704,0.132808 0.255853,0.179179 0.316164,-0.106107 -0.128409,-0.263774 -0.020646,0.154569 0.331313,0.043794 0.082853,-0.323463 -0.299623,-0.132191 -0.171279,0.140744 -0.339829,-0.002229

Powellvenator_podocitus_PVL_441 0.274049,0.109793 -0.286370,0.133298 0.273957,0.109209 0.305314,-0.057318 -0.097901,-0.182064 -0.013047,0.152132 0.251116,0.014580 0.196499,-0.332544 -0.365795,-0.145430 -0.159883,0.194597 -0.396512,-0.039352

Proterosuchus_alexanderi_NMQR_1 0.210078,0.172914 -0.284290,0.123406 0.317538,0.153504 0.385752,-0.092718 -0.075469,-0.181133 -0.040257,0.090390 0.366190,0.013772 0.044159,-0.249841 -0.331311,-0.128000 -0.151647,0.077839 -0.421309,0.004180

Proterosuchus_sp_AMNH_2237_espe 0.224797,0.141799 -0.347769,0.127901 0.336137,0.135802 0.387146,-0.084826 -0.092360,-0.177972 -0.045311,0.080906 0.367717,0.024170 0.066403,-0.219766 -0.315040,-0.107907 -0.195615,0.072488 -0.389910,0.019968

Rauisuchus_tiradentes_espejada 0.224478,0.148693 -0.255304,0.190873 0.224786,0.148626 0.381573,-0.048300 -0.094784,-0.313863 -0.018709,0.103204 0.308498,0.053027 0.069008,-0.302004 -0.343454,-0.156913 -0.137802,0.156779 -0.361623,0.020851

Riojasuchus_tenuisceps_PVL_3827 0.260512,0.248018 -0.201581,0.161477 0.260488,0.247903 0.343900,-0.089892 -0.132921,-0.367525 0.037918,0.115204 0.190490,0.041044 0.041991,-0.333018 -0.391049,-0.154786 -0.088159,0.106640 -0.341493,-0.003755

Saturnalia_tupiniquim_MCP_3844_ 0.284126,0.164898 -0.301293,0.153822 0.283554,0.165057 0.304358,-0.126375 -0.068796,-0.204302 -0.025861,0.100899 0.245468,-0.002034 0.186968,-0.270088 -0.364132,-0.128340 -0.173491,0.122137 -0.395187,0.005283

Shansisuchus_shansisuchus 0.073121,0.108652 -0.291303,0.166813 0.329154,0.091143 0.404091,-0.067008 -0.068561,-0.192607 -0.110687,0.090230 0.373162,0.015463 0.198289,-0.315065 -0.325476,-0.084735 -0.172887,0.128508 -0.386949,0.049080

Shringasaurus 0.222709,0.174819 -0.333736,0.091686 0.351905,0.134850 0.365150,-0.105185 -0.081883,-0.177031 -0.057815,0.150271 0.372526,0.006311 0.039647,-0.238738 -0.301241,-0.108831 -0.183639,0.100001 -0.386915,-0.014208

Silesaurus_opolensis_ZPAL_Ab_II 0.226817,0.152558 -0.322060,0.154531 0.227213,0.152472 0.371716,-0.153929 -0.057059,-0.173775 -0.054559,0.131086 0.263344,-0.029825 0.225695,-0.320835 -0.283117,-0.089585 -0.191462,0.142546 -0.407509,0.022676

Smilosuchus_gregorii_USNM_18313 0.288871,0.127548 -0.376117,0.129536 0.289960,0.127195 0.382704,-0.081271 -0.074494,-0.191704 -0.044755,0.102564 0.307914,0.008160 0.092692,-0.234465 -0.276290,-0.095290 -0.218126,0.126708 -0.397874,0.015002

Tawa_hallae_GR_242_espejada 0.218756,0.148870 -0.275125,0.186558 0.218008,0.147639 0.348527,-0.138924 -0.065444,-0.201954 -0.038242,0.096623 0.238228,-0.013936 0.279379,-0.297572 -0.354013,-0.118411 -0.155897,0.145386 -0.421870,0.040942

Vancleavea_campi 0.097012,0.236481 -0.269389,0.126102 0.301312,0.131852 0.368694,-0.172885 -0.080306,-0.211851 -0.094402,0.193074 0.342392,-0.019588 0.154211,-0.342915 -0.283265,-0.102984 -0.163469,0.157727 -0.359845,0.004479

Pamelaria_dolichotrachela_ISIR_ 0.188763,0.173156 -0.362958,0.141768 0.329071,0.131716 0.353628,-0.093965 -0.022184,-0.214567 0.029801,0.131645 0.347469,0.005201 0.017885,-0.238162 -0.283985,-0.136855 -0.139572,0.133716 -0.439072,-0.008973

;

tread

(((Shansisuchus_shansisuchus (Euparkeria_capensis_UMZC_T692 (((Marasuchus_lilloensis ((((Eoraptor_lunensis_espejada (Saturnalia_tupiniquim_MCP_3844_ (Jaklapallisaurus_assymetrica_IS (Adeopapposaurus_mognai_PVSJ_610 Plateosaurus_engelhardti_SMNS_1))))(Herrerasaurus_ischigualastensis (Tawa_hallae_GR_242_espejada ((Liliensternus_liliensterni_MB_R (Cryolophosaurus_ellioti (Ceratosaurus_nasicornis_USNM_47 Dilophosaurus_wetherilli_UCMP_3)))(Megapnosaurus_rhodesiensis_QR_6 Powellvenator_podocitus_PVL_441)))))(Pisanosaurus_mertii_PVL_2577 Sctuellosaurus_lawleri_UCMP_130))(Lewisuchus_admixtus_MACN_Pv (Asilisaurus_kongwe_1 Silesaurus_opolensis_ZPAL_Ab_II))))((Nundasuchus_songeaensis ((Riojasuchus_tenuisceps_PVL_3827 (Aetosaurus_ferratus Polesinesuchus_aurelioi))(Gracilisuchus_stipanicicorum_PV (Effigia_okeeffeae_espejada (Mandasuchus_tanyauchen (Fasolasuchus_tenax_PVL_3850 (Rauisuchus_tiradentes_espejada (Postosuchus_alisonae_espejada Postosuchus_kirkpatricki))))))))(Parasuchus_hislopi_ISI_R_espeja (Pseudopalatus_UCMP_27235_espeja Smilosuchus_gregorii_USNM_18313))))(Rhadinosuchinae_espejada Vancleavea_campi))))(Proterosuchus_alexanderi_NMQR_1 Proterosuchus_sp_AMNH_2237_espe))(Pamelaria_dolichotrachela_ISIR_ (Azendohsaurus_madagaskarensis Shringasaurus)))

;

proc/;

Phylogenetic matrix of the centrale loss hypothesis in Ezcurra (2016) topology

xread

'Landmark data, imported from TPS to TNT' 1 42

&[landmark 2d]

Gracilisuchus_stipanicicorum_PV 0.281627,0.144227 -0.284647,0.125547 0.281343,0.143378 0.317194,-0.034097 -0.145625,-0.244464 -0.022380,0.114205 0.312142,0.059012 0.107588,-0.326506 -0.335813,-0.111131 -0.164330,0.122926 -0.367543,0.010762

Pseudopalatus_UCMP_27235_espeja 0.212767,0.170726 -0.274401,0.160618 0.212944,0.170694 0.378545,-0.096750 -0.121900,-0.275556 -0.026534,0.119184 0.304656,0.043708 0.108648,-0.359507 -0.294795,-0.112865 -0.154452,0.151046 -0.348811,0.032201

Rhadinosuchinae_espejada 0.236826,0.158787 -0.277352,0.172569 0.236776,0.158796 0.363617,-0.098992 -0.085885,-0.238872 -0.039033,0.110198 0.282392,0.007281 0.143415,-0.307169 -0.320235,-0.124577 -0.163333,0.131285 -0.394887,0.035728

Asilisaurus_kongwe_1 0.233277,0.125927 -0.309152,0.179166 0.233721,0.125845 0.372913,-0.094040 -0.076138,-0.197311 -0.034461,0.078145 0.278740,-0.022515 0.239895,-0.273704 -0.336396,-0.095147 -0.178800,0.114468 -0.424748,0.057220

Sctuellosaurus_lawleri_UCMP_130 0.239904,0.067952 -0.318716,0.188384 0.240341,0.067872 0.372124,-0.016775 -0.066427,-0.216122 -0.036011,0.122310 0.310199,0.015039 0.164518,-0.287067 -0.319434,-0.133732 -0.176989,0.174830 -0.422857,0.025154

Effigia_okeeffeae_espejada 0.182345,0.178971 -0.220069,0.201566 0.183136,0.178826 0.351803,-0.068310 -0.082156,-0.351278 -0.015689,0.121270 0.289250,0.068606 0.119191,-0.418820 -0.337348,-0.114869 -0.130944,0.133773 -0.336045,0.070728

Fasolasuchus_tenax_PVL_3850 0.254265,0.200752 -0.216240,0.178573 0.254950,0.200207 0.279143,-0.020642 -0.122268,-0.346656 0.034200,0.091353 0.227556,0.078524 0.125122,-0.416139 -0.380212,-0.120138 -0.095266,0.128044 -0.346266,0.036589

Adeopapposaurus_mognai_PVSJ_610 0.283692,0.126786 -0.316474,0.145472 0.284640,0.126119 0.306844,-0.092344 -0.088678,-0.167994 -0.014837,0.079818 0.287588,-0.000718 0.237578,-0.251233 -0.342905,-0.097458 -0.171508,0.084448 -0.462425,0.039666

Aetosaurus_ferratus 0.221581,0.210551 -0.211015,0.152956 0.222382,0.210404 0.305571,-0.078519 -0.109133,-0.348687 0.003008,0.168966 0.287929,0.059898 0.041634,-0.414363 -0.343149,-0.146831 -0.110082,0.167737 -0.320190,-0.001651

Azendohsaurus_madagaskarensis 0.143080,0.192171 -0.310238,0.098427 0.316903,0.187905 0.417471,-0.153606 -0.108822,-0.275783 -0.098052,0.194124 0.375980,0.015468 -0.008518,-0.308799 -0.233346,-0.102606 -0.204642,0.160859 -0.286247,0.006301

Ceratosaurus_nasicornis_USNM_47 0.261498,0.187141 -0.270358,0.118992 0.261725,0.187099 0.303617,-0.120156 -0.110783,-0.181675 -0.001955,0.131460

0.243129,0.014938 0.174101,-0.380603 -0.333501,-0.117920 -0.137843,0.143533 -0.396768,-0.006226

Cryolophosaurus_ellioti 0.277272,0.186484 -0.280524,0.142789 0.277411,0.186458 0.319525,-0.139186 -0.083636,-0.189897 0.003472,0.098620 0.226214,0.015100 0.153475,-0.312798 -0.376143,-0.136180 -0.140973,0.130004 -0.389330,-0.011649

Dilophosaurus_wetherilli_UCMP_3 0.286929,0.184820 -0.289907,0.128567 0.287185,0.184773 0.305264,-0.128386 -0.100725,-0.177491 0.000879,0.124182 0.228293,0.001288 0.168975,-0.320763 -0.348184,-0.130305 -0.147831,0.138816 -0.394871,-0.014166

Eoraptor_lunensis_espejada 0.293512,0.136973 -0.311023,0.161697 0.293721,0.136935 0.327367,-0.098853 -0.078256,-0.178800 -0.018819,0.098488 0.241651,-0.000724 0.180803,-0.256737 -0.366649,-0.140979 -0.172378,0.125058 -0.409480,0.004823

Euparkeria_capensis_UMZC_T692 0.208910,0.130503 -0.297184,0.161471 0.208463,0.129220 0.398939,-0.113516 -0.095902,-0.245639 -0.044215,0.131360 0.325044,0.024742 0.145501,-0.296298 -0.298354,-0.108318 -0.175061,0.153447 -0.381771,0.032602

Herrerasaurus_ischigualastensis 0.267866,0.127600 -0.326118,0.187512 0.268520,0.127912 0.332130,-0.141010 -0.049158,-0.162452 -0.066208,0.115211 0.260220,-0.029024 0.246171,-0.254488 -0.328454,-0.125992 -0.209762,0.139441 -0.405494,0.029637

Jaklapallisaurus_assymetrica_IS 0.264736,0.142222 -0.309540,0.151551 0.264280,0.141095 0.334698,-0.097612 -0.076710,-0.207651 -0.019334,0.108951 0.265680,0.003303 0.185390,-0.283588 -0.345914,-0.109852 -0.168206,0.142320 -0.402301,0.020108

Lewisuchus_admixtus_MACN_Pv 0.226990,0.127344 -0.278375,0.192398 0.227016,0.127776 0.367466,-0.075854 -0.047081,-0.225587 -0.028929,0.083629 0.271638,-0.002525 0.210257,-0.279211 -0.386934,-0.107402 -0.164787,0.116797 -0.414807,0.058931

Liliensternus_liliensterni_MB_R 0.248183,0.156593 -0.282460,0.160307 0.248437,0.157003 0.340514,-0.121461 -0.066144,-0.178175 -0.027318,0.145170 0.237025,-0.012101 0.181550,-0.335737 -0.351342,-0.139489 -0.158521,0.153428 -0.392726,-0.001150

Mandasuchus_tanyauchen 0.259413,0.174613 -0.280897,0.147592 0.259414,0.174613 0.343364,-0.107083 -0.104694,-0.267950 -0.027807,0.140242 0.273198,0.019960 0.095138,-0.311609 -0.321149,-0.136955 -0.162648,0.156987 -0.350610,0.001346

Marasuchus_lilloensis 0.206915,0.154796 -0.238493,0.186270 0.207231,0.154738 0.418496,-0.078029 -0.103648,-0.260313 -0.027948,0.086034 0.262266,-0.009019 0.172851,-0.293661 -0.369736,-0.125641 -0.136124,0.126961 -0.392277,0.050311

Megapnosaurus_rhodesiensis_QR_6 0.206759,0.117267 -0.297386,0.155467 0.207035,0.117216 0.390752,-0.109596 -0.069623,-0.196448 -0.046037,0.167446 0.295361,0.002623 0.165794,-0.346097 -0.317395,-0.124726 -0.180069,0.202425 -0.366336,-0.002041

Nundasuchus_songeaensis 0.243699,0.193945 -0.265998,0.153038 0.243783,0.193930 0.343673,-0.139308 -0.100046,-0.265755 -0.014015,0.128816 0.280291,0.007031 0.113311,-0.285614 -0.327282,-0.138125 -0.142509,0.137017 -0.382579,0.014372

Parasuchus_hislopi_ISI_R_espeja 0.239130,0.183564 -0.274462,0.157392 0.238828,0.183682 0.364984,-0.112461 -0.084067,-0.290198 -0.026751,0.129070 0.291273,0.022400 0.076912,-0.303434 -0.323967,-0.124069 -0.158194,0.132259 -0.355371,0.025863

Pisanosaurus_mertii_PVL_2577 0.308017,0.120875 -0.296257,0.113480 0.307243,0.121200 0.288345,-0.055140 -0.107411,-0.171571 0.007744,0.108387 0.306925,0.030652 0.131195,-0.259524 -0.420042,-0.137937 -0.154203,0.122110 -0.405549,-0.043032

Plateosaurus_engelhardti_SMNS_1 0.310825,0.100117 -0.314663,0.151792 0.310467,0.100217 0.281367,-0.037420 -0.103589,-0.161789 -0.021664,0.085503 0.290684,0.027984 0.219330,-0.268029 -0.352106,-0.121236 -0.175515,0.111117 -0.446165,0.020856

Polesinesuchus_aurelioi 0.276099,0.183361 -0.280697,0.120245 0.276387,0.183308 0.351313,-0.103105 -0.105012,-0.253627 -0.008803,0.125561 0.313498,0.036346 0.017949,-0.274547 -0.329119,-0.143162 -0.151285,0.143519 -0.367763,-0.019188

Postosuchus_alisonae_espejada 0.265634,0.194667 -0.243561,0.143747 0.266061,0.194588 0.294109,-0.056303 -0.153312,-0.319314 0.014471,0.125097 0.269655,0.064638 0.091190,-0.360796 -0.339283,-0.132076 -0.120366,0.126186 -0.352169,0.016881

Postosuchus_kirkpatricki 0.256315,0.177575 -0.294528,0.134220 0.256461,0.177548 0.315074,-0.107739 -0.129788,-0.262756 -0.019763,0.154521 0.331138,0.041927 0.080824,-0.323516 -0.299858,-0.130330 -0.169120,0.141594 -0.339169,0.002201

Powellvenator_podocitus_PVL_441 0.275066,0.108858 -0.286325,0.134983 0.275058,0.108265 0.305819,-0.059007 -0.099041,-0.182153 -0.012398,0.152479 0.251830,0.013421 0.195155,-0.334218 -0.367202,-0.143923 -0.157904,0.195769 -0.395713,-0.031433

Proterosuchus_alexanderi_NMQR_1 0.179772,0.218878 -0.289475,0.088069 0.313349,0.198426 0.406080,-0.104975 -0.163362,-0.229980 -0.039667,0.113611 0.378130,0.026624 -0.012081,-0.311477 -0.285958,-0.101075 -0.159985,0.110679 -0.326469,-0.008340

Proterosuchus_sp_AMNH_2237_espe 0.198896,0.181735 -0.293065,0.075785 0.336764,0.176871 0.405097,-0.095601 -0.187042,-0.222107 -0.047813,0.126599 0.378406,0.039109 0.010896,-0.270493 -0.309479,-0.102276 -0.167915,0.103045 -0.326313,-0.018244

Rauisuchus_tiradentes_espejada 0.225296,0.147089 -0.253767,0.192490 0.225623,0.147029 0.381034,-0.050872 -0.096965,-0.313132 -0.018020,0.103281 0.308681,0.050912 0.066791,-0.302356 -0.344279,-0.154454 -0.136425,0.157576 -0.359990,0.027580

Riojasuchus_tenuisceps_PVL_3827 0.261402,0.246524 -0.200210,0.162268 0.261457,0.246432 0.343025,-0.091681 -0.135068,-0.366732 0.038462,0.114926 0.190366,0.040031 0.039828,-0.333099 -0.391348,-0.152435 -0.086432,0.106806 -0.337626,0.002967

Saturnalia_tupiniquim_MCP_3844_ 0.285573,0.163540 -0.300938,0.155912 0.285069,0.163692 0.304314,-0.128384 -0.070224,-0.204379 -0.025322,0.101306 0.246044,-0.003457 0.185613,-0.271843 -0.365558,-0.126410 -0.172200,0.123277 -0.394957,0.011743

Shansisuchus_shansisuchus 0.073846,0.108120 -0.289600,0.169218 0.329831,0.088342 0.403463,-0.070604 -0.070527,-0.192185 -0.110007,0.091274 0.373236,0.012203 0.195263,-0.317001 -0.325998,-0.081750 -0.174273,0.131215 -0.385585,0.054984

Shringasaurus 0.185949,0.200061 -0.293733,0.078259 0.339722,0.163874 0.373895,-0.114509 -0.140757,-0.233220 -0.065131,0.176005 0.373827,0.015938 0.005246,-0.295630 -0.293926,-0.115977 -0.179159,0.137250 -0.318473,-0.029550

Silesaurus_opolensis_ZPAL_Ab_II 0.228190,0.150970 -0.321049,0.157182 0.228662,0.150883 0.370854,-0.156986 -0.058644,-0.173646 -0.053593,0.131672 0.263396,-0.031874 0.223197,-0.323029 -0.284098,-0.087429 -0.190140,0.144187 -0.407756,0.027547

Smilosuchus_gregorii_USNM_18313 0.289977,0.125859 -0.375576,0.132200 0.291128,0.125495 0.382613,-0.083947 -0.075951,-0.191554 -0.044133,0.103034 0.308336,0.006145 0.091257,-0.235527 -0.277313,-0.093588 -0.217746,0.128367 -0.398245,0.017916

Tawa_hallae_GR_242_espejada 0.220402,0.147525 -0.274185,0.189045 0.219761,0.146297 0.348202,-0.141954 -0.067399,-0.202045 -0.037577,0.097237 0.238765,-0.015788 0.277191,-0.300612 -0.355641,-0.115799 -0.154536,0.146751 -0.421031,0.048660

Vancleavea_campi 0.098081,0.235642 -0.267921,0.127575 0.301706,0.129644 0.366945,-0.175257 -0.081803,-0.211080 -0.093131,0.193568 0.341760,-0.021911 0.151489,-0.343617 -0.283282,-0.100786 -0.162746,0.158389 -0.358645,0.009217

Pamelaria_dolichotrachela_ISIR_ 0.136529,0.218546 -0.265453,0.137665 0.320318,0.186925 0.383908,-0.094976 -0.073590,-0.301692 -0.057982,0.143446 0.361899,0.029673 -0.020135,-0.326346 -0.296651,-0.113425 -0.144731,0.121086 -0.333133,0.023387

;

tread

(((Shansisuchus_shansisuchus (Euparkeria_capensis_UMZC_T692 (((Marasuchus_lilloensis ((((Eoraptor_lunensis_espejada (Saturnalia_tupiniquim_MCP_3844_ (Jaklapallisaurus_assymetrica_IS (Adeopapposaurus_mognai_PVSJ_610 Plateosaurus_engelhardti_SMNS_1))))(Herrerasaurus_ischigualastensis (Tawa_hallae_GR_242_espejada ((Liliensternus_liliensterni_MB_R (Cryolophosaurus_ellioti (Ceratosaurus_nasicornis_USNM_47 Dilophosaurus_wetherilli_UCMP_3)))(Megapnosaurus_rhodesiensis_QR_6 Powellvenator_podocitus_PVL_441)))))(Pisanosaurus_mertii_PVL_2577 Sctuellosaurus_lawleri_UCMP_130))(Lewisuchus_admixtus_MACN_Pv (Asilisaurus_kongwe_1 Silesaurus_opolensis_ZPAL_Ab_II))))((Nundasuchus_songeaensis ((Riojasuchus_tenuisceps_PVL_3827 (Aetosaurus_ferratus Polesinesuchus_aurelioi))(Gracilisuchus_stipanicicorum_PV (Effigia_okeeffeae_espejada (Mandasuchus_tanyauchen (Fasolasuchus_tenax_PVL_3850 (Rauisuchus_tiradentes_espejada (Postosuchus_alisonae_espejada Postosuchus_kirkpatricki))))))))(Parasuchus_hislopi_ISI_R_espeja (Pseudopalatus_UCMP_27235_espeja Smilosuchus_gregorii_USNM_18313))))(Rhadinosuchinae_espejada Vancleavea_campi))))(Proterosuchus_alexanderi_NMQR_1 Proterosuchus_sp_AMNH_2237_espe))(Pamelaria_dolichotrachela_ISIR_ (Azendohsaurus_madagaskarensis Shringasaurus)))

;

proc/;

Phylogenetic matrix of the centrale+astragalus hypothesis in Nesbitt (2011) topology

xread

'Landmark data, imported from TPS to TNT' 1 42

&[landmark 2d]

Gracilisuchus_stipanicicorum_PV 0.280821,0.145902 -0.285444,0.123894 0.280533,0.145049 0.317436,-0.032226 -0.144156,-0.245331 -0.023047,0.114079 0.311828,0.060858 0.109562,-0.325880 -0.335212,-0.113146 -0.166368,0.122012 -0.368342,0.005558

Pseudopalatus_UCMP_27235_espeja 0.211945,0.172573 -0.276128,0.158861 0.212150,0.172528 0.379872,-0.094089 -0.119957,-0.276830 -0.027447,0.119186 0.304839,0.046001 0.111602,-0.359192 -0.294566,-0.115293 -0.156408,0.150214 -0.349974,0.027174

Rhadinosuchinae_espejada 0.235592,0.160439 -0.278485,0.170515 0.235539,0.160451 0.364209,-0.096346 -0.084149,-0.239420 -0.039822,0.109879 0.282239,0.009307 0.145566,-0.306042 -0.319216,-0.126833 -0.164667,0.130178 -0.395435,0.029614

Asilisaurus_kongwe_1 0.231766,0.127483 -0.310086,0.176288 0.232105,0.127409 0.372916,-0.090689 -0.074179,-0.197355 -0.035012,0.077561 0.278289,-0.020235 0.241960,-0.270923 -0.334919,-0.097730 -0.179732,0.112767 -0.424789,0.051072

Sctuellosaurus_lawleri_UCMP_130 0.238992,0.069362 -0.319598,0.185766 0.239329,0.069289 0.371529,-0.013816 -0.064540,-0.216065 -0.036820,0.121771 0.309502,0.017282 0.166444,-0.285144 -0.317827,-0.135881 -0.178268,0.173294 -0.422423,0.018328

Effigia_okeeffeae_espejada 0.181292,0.180946 -0.222711,0.199904 0.182057,0.180779 0.353166,-0.065158 -0.078814,-0.352378 -0.016806,0.121325 0.289215,0.071344 0.123602,-0.418169 -0.337081,-0.118386 -0.132987,0.132965 -0.338961,0.064572

Fasolasuchus_tenax_PVL_3850 0.253114,0.202693 -0.217987,0.177191 0.253711,0.202127 0.279551,-0.018580 -0.119446,-0.347555 0.033602,0.091643 0.227199,0.080210 0.128585,-0.415215 -0.379719,-0.123201 -0.097271,0.127710 -0.349651,0.029666

Adeopapposaurus_mognai_PVSJ_610 0.282068,0.128194 -0.316618,0.142764 0.282860,0.127541 0.306283,-0.089810 -0.087015,-0.167710 -0.015251,0.079255 0.286593,0.001246 0.239009,-0.248352 -0.341142,-0.099530 -0.172336,0.082893 -0.461887,0.033896

Aetosaurus_ferratus 0.221016,0.212191 -0.212754,0.152141 0.221797,0.212021 0.306866,-0.076872 -0.107038,-0.349739 0.002010,0.169220 0.288269,0.061648 0.044622,-0.414676 -0.343177,-0.149268 -0.113047,0.167232 -0.323365,-0.007945

Azendohsaurus_madagaskarensis 0.155484,0.177603 -0.253514,0.099892 0.313627,0.170727 0.398476,-0.139323 -0.077993,-0.240054 -0.061205,0.183948 0.364266,0.014158 0.010045,-0.270762 -0.293749,-0.138597 -0.142012,0.162252 -0.393935,-0.029348

Ceratosaurus_nasicornis_USNM_47 0.260384,0.188426 -0.270859,0.117473 0.260571,0.188385 0.304046,-0.118458 -0.109695,-0.182088 -0.002623,0.131361

0.242874,0.016225 0.176105,-0.379435 -0.332696,-0.119656 -0.139719,0.142637 -0.397752,-0.012797

Cryolophosaurus_ellioti 0.275981,0.187556 -0.280762,0.141179 0.276061,0.187539 0.319617,-0.137460 -0.082524,-0.189895 0.003046,0.098426 0.225654,0.016171 0.154799,-0.311508 -0.374933,-0.137823 -0.142281,0.129222 -0.390423,-0.018736

Dilophosaurus_wetherilli_UCMP_3 0.285762,0.185923 -0.290150,0.127015 0.285954,0.185881 0.305374,-0.126803 -0.099699,-0.177596 0.000354,0.124000 0.227881,0.002331 0.170343,-0.319486 -0.347134,-0.131795 -0.149347,0.137927 -0.395391,-0.020690

Eoraptor_lunensis_espejada 0.292140,0.138129 -0.311161,0.159598 0.292255,0.138104 0.326955,-0.096790 -0.076981,-0.178565 -0.019271,0.098043 0.240865,0.000547 0.181875,-0.254873 -0.364989,-0.142673 -0.173346,0.123883 -0.409339,-0.001970

Euparkeria_capensis_UMZC_T692 0.207978,0.132115 -0.298494,0.159237 0.207528,0.130824 0.399862,-0.110467 -0.093993,-0.246380 -0.045208,0.131030 0.324902,0.027230 0.147825,-0.295200 -0.297603,-0.110638 -0.176826,0.152184 -0.382149,0.027470

Herrerasaurus_ischigualastensis 0.266417,0.129006 -0.326755,0.184869 0.266941,0.129329 0.332181,-0.138401 -0.047648,-0.162082 -0.066896,0.114386 0.259654,-0.027259 0.247691,-0.251830 -0.326746,-0.128018 -0.210728,0.137688 -0.404895,0.023433

Jaklapallisaurus_assymetrica_IS 0.263404,0.143692 -0.310070,0.149254 0.262899,0.142569 0.334747,-0.095232 -0.075158,-0.207747 -0.020002,0.108611 0.265164,0.005028 0.187052,-0.281820 -0.344630,-0.111940 -0.169683,0.141082 -0.402275,0.013961

Lewisuchus_admixtus_MACN_Pv 0.225396,0.128774 -0.279277,0.189770 0.225322,0.129215 0.367206,-0.072601 -0.044945,-0.225344 -0.029521,0.083074 0.270934,-0.000356 0.212246,-0.276670 -0.385253,-0.110285 -0.165831,0.115305 -0.415933,0.051264

Liliensternus_liliensterni_MB_R 0.246947,0.157768 -0.282966,0.158476 0.247151,0.158186 0.340692,-0.119370 -0.064973,-0.178199 -0.028103,0.144830 0.236666,-0.010774 0.183283,-0.334230 -0.350060,-0.141323 -0.160164,0.152369 -0.393323,-0.008515

Mandasuchus_tanyauchen 0.258585,0.176211 -0.281947,0.146094 0.258605,0.176206 0.344226,-0.105186 -0.103224,-0.268712 -0.028627,0.140169 0.273291,0.021536 0.097020,-0.311282 -0.320606,-0.138881 -0.164599,0.156176 -0.351389,-0.004279

Marasuchus_lilloensis 0.205578,0.156204 -0.239717,0.184354 0.205839,0.156147 0.418779,-0.074740 -0.101504,-0.260843 -0.028549,0.085696 0.262086,-0.007044 0.175031,-0.292011 -0.368528,-0.128384 -0.137333,0.125968 -0.394377,0.042652

Megapnosaurus_rhodesiensis_QR_6 0.205991,0.118544 -0.298372,0.153536 0.206233,0.118491 0.391376,-0.107030 -0.068261,-0.196795 -0.047089,0.167101 0.295280,0.004524 0.168059,-0.344895 -0.316549,-0.126779 -0.182273,0.201163 -0.366632,-0.008722

Nundasuchus_songeaensis 0.242588,0.195397 -0.266947,0.151488 0.242666,0.195380 0.344533,-0.137277 -0.098459,-0.266354 -0.014772,0.128739 0.280280,0.008691 0.115034,-0.284957 -0.326509,-0.140083 -0.144202,0.136261 -0.383496,0.008409

Parasuchus_hislopi_ISI_R_espeja 0.238135,0.185307 -0.275797,0.155702 0.237848,0.185416 0.366060,-0.110097 -0.082183,-0.290957 -0.027632,0.128997 0.291393,0.024366 0.079092,-0.303183 -0.323458,-0.126364 -0.159929,0.131396 -0.356432,0.020584

Pisanosaurus_mertii_PVL_2577 0.306788,0.121378 -0.295686,0.111931 0.305898,0.121718 0.287315,-0.053787 -0.106444,-0.171107 0.007471,0.107979 0.305802,0.031662 0.131667,-0.257955 -0.418414,-0.139000 -0.155686,0.121103 -0.406929,-0.050219

Plateosaurus_engelhardti_SMNS_1 0.309466,0.101402 -0.314687,0.149381 0.308939,0.101508 0.280336,-0.035319 -0.102096,-0.161476 -0.022030,0.084893 0.289476,0.029693 0.220628,-0.265438 -0.350255,-0.123033 -0.176607,0.109730 -0.445682,0.014408

Polesinesuchus_aurelioi 0.275458,0.184553 -0.281352,0.119149 0.275725,0.184495 0.351866,-0.101646 -0.103901,-0.254095 -0.009312,0.125547 0.313450,0.037664 0.019213,-0.274519 -0.328675,-0.144618 -0.152872,0.142895 -0.368649,-0.024660

Postosuchus_alisonae_espejada 0.264814,0.196466 -0.244884,0.142493 0.265231,0.196376 0.294899,-0.054599 -0.151351,-0.320471 0.013712,0.125314 0.269635,0.066305 0.093673,-0.360538 -0.338961,-0.134362 -0.122648,0.125689 -0.354142,0.011251

Postosuchus_kirkpatricki 0.255684,0.179216 -0.295704,0.132808 0.255853,0.179179 0.316164,-0.106107 -0.128409,-0.263774 -0.020646,0.154569 0.331313,0.043794 0.082853,-0.323463 -0.299623,-0.132191 -0.171279,0.140744 -0.339829,-0.002229

Powellvenator_podocitus_PVL_441 0.274049,0.109793 -0.286370,0.133298 0.273957,0.109209 0.305314,-0.057318 -0.097901,-0.182064 -0.013047,0.152132 0.251116,0.014580 0.196499,-0.332544 -0.365795,-0.145430 -0.159883,0.194597 -0.396512,-0.039352

Proterosuchus_alexanderi_NMQR_1 0.210078,0.172914 -0.284290,0.123406 0.317538,0.153504 0.385752,-0.092718 -0.075469,-0.181133 -0.040257,0.090390 0.366190,0.013772 0.044159,-0.249841 -0.331311,-0.128000 -0.151647,0.077839 -0.421309,0.004180

Proterosuchus_sp_AMNH_2237_espe 0.224797,0.141799 -0.347769,0.127901 0.336137,0.135802 0.387146,-0.084826 -0.092360,-0.177972 -0.045311,0.080906 0.367717,0.024170 0.066403,-0.219766 -0.315040,-0.107907 -0.195615,0.072488 -0.389910,0.019968

Rauisuchus_tiradentes_espejada 0.224478,0.148693 -0.255304,0.190873 0.224786,0.148626 0.381573,-0.048300 -0.094784,-0.313863 -0.018709,0.103204 0.308498,0.053027 0.069008,-0.302004 -0.343454,-0.156913 -0.137802,0.156779 -0.361623,0.020851

Riojasuchus_tenuisceps_PVL_3827 0.260512,0.248018 -0.201581,0.161477 0.260488,0.247903 0.343900,-0.089892 -0.132921,-0.367525 0.037918,0.115204 0.190490,0.041044 0.041991,-0.333018 -0.391049,-0.154786 -0.088159,0.106640 -0.341493,-0.003755

Saturnalia_tupiniquim_MCP_3844_ 0.284126,0.164898 -0.301293,0.153822 0.283554,0.165057 0.304358,-0.126375 -0.068796,-0.204302 -0.025861,0.100899 0.245468,-0.002034 0.186968,-0.270088 -0.364132,-0.128340 -0.173491,0.122137 -0.395187,0.005283

Shansisuchus_shansisuchus 0.073121,0.108652 -0.291303,0.166813 0.329154,0.091143 0.404091,-0.067008 -0.068561,-0.192607 -0.110687,0.090230 0.373162,0.015463 0.198289,-0.315065 -0.325476,-0.084735 -0.172887,0.128508 -0.386949,0.049080

Shringasaurus 0.222709,0.174819 -0.333736,0.091686 0.351905,0.134850 0.365150,-0.105185 -0.081883,-0.177031 -0.057815,0.150271 0.372526,0.006311 0.039647,-0.238738 -0.301241,-0.108831 -0.183639,0.100001 -0.386915,-0.014208

Silesaurus_opolensis_ZPAL_Ab_II 0.226817,0.152558 -0.322060,0.154531 0.227213,0.152472 0.371716,-0.153929 -0.057059,-0.173775 -0.054559,0.131086 0.263344,-0.029825 0.225695,-0.320835 -0.283117,-0.089585 -0.191462,0.142546 -0.407509,0.022676

Smilosuchus_gregorii_USNM_18313 0.288871,0.127548 -0.376117,0.129536 0.289960,0.127195 0.382704,-0.081271 -0.074494,-0.191704 -0.044755,0.102564 0.307914,0.008160 0.092692,-0.234465 -0.276290,-0.095290 -0.218126,0.126708 -0.397874,0.015002

Tawa_hallae_GR_242_espejada 0.218756,0.148870 -0.275125,0.186558 0.218008,0.147639 0.348527,-0.138924 -0.065444,-0.201954 -0.038242,0.096623 0.238228,-0.013936 0.279379,-0.297572 -0.354013,-0.118411 -0.155897,0.145386 -0.421870,0.040942

Vancleavea_campi 0.097012,0.236481 -0.269389,0.126102 0.301312,0.131852 0.368694,-0.172885 -0.080306,-0.211851 -0.094402,0.193074 0.342392,-0.019588 0.154211,-0.342915 -0.283265,-0.102984 -0.163469,0.157727 -0.359845,0.004479

Pamelaria_dolichotrachela_ISIR_ 0.188763,0.173156 -0.362958,0.141768 0.329071,0.131716 0.353628,-0.093965 -0.022184,-0.214567 0.029801,0.131645 0.347469,0.005201 0.017885,-0.238162 -0.283985,-0.136855 -0.139572,0.133716 -0.439072,-0.008973

;

tread

(((Shansisuchus_shansisuchus (Vancleavea_campi (Rhadinosuchinae_espejada (Euparkeria_capensis_UMZC_T692 (((Marasuchus_lilloensis ((((Eoraptor_lunensis_espejada (Saturnalia_tupiniquim_MCP_3844_ (Jaklapallisaurus_assymetrica_IS (Adeopapposaurus_mognai_PVSJ_610 Plateosaurus_engelhardti_SMNS_1))))(Herrerasaurus_ischigualastensis (Tawa_hallae_GR_242_espejada ((Liliensternus_liliensterni_MB_R (Cryolophosaurus_ellioti (Ceratosaurus_nasicornis_USNM_47 Dilophosaurus_wetherilli_UCMP_3)))(Megapnosaurus_rhodesiensis_QR_6 Powellvenator_podocitus_PVL_441)))))(Pisanosaurus_mertii_PVL_2577 Sctuellosaurus_lawleri_UCMP_130))(Lewisuchus_admixtus_MACN_Pv (Asilisaurus_kongwe_1 Silesaurus_opolensis_ZPAL_Ab_II))))(Riojasuchus_tenuisceps_PVL_3827 ((Aetosaurus_ferratus Polesinesuchus_aurelioi)(Gracilisuchus_stipanicicorum_PV (Nundasuchus_songeaensis (Effigia_okeeffeae_espejada (Mandasuchus_tanyauchen (Fasolasuchus_tenax_PVL_3850 (Rauisuchus_tiradentes_espejada (Postosuchus_alisonae_espejada Postosuchus_kirkpatricki))))))))(Parasuchus_hislopi_ISI_R_espeja (Pseudopalatus_UCMP_27235_espeja Smilosuchus_gregorii_USNM_18313)))))))(Proterosuchus_alexanderi_NMQR_1 Proterosuchus_sp_AMNH_2237_espe))(Pamelaria_dolichotrachela_ISIR_ (Azendohsaurus_madagaskarensis Shringasaurus)))

```
;
proc/;
```

Phylogenetic matrix of the centrale loss hypothesis in Nesbitt (2011) topology

xread

'Landmark data, imported from TPS to TNT' 1 42

&[landmark 2d]

Gracilisuchus_stipanicicorum_PV 0.281627,0.144227 -0.284647,0.125547 0.281343,0.143378 0.317194,-0.034097 -0.145625,-0.244464 -0.022380,0.114205 0.312142,0.059012 0.107588,-0.326506 -0.335813,-0.111131 -0.164330,0.122926 -0.367543,0.010762

Pseudopalatus_UCMP_27235_espeja 0.212767,0.170726 -0.274401,0.160618 0.212944,0.170694 0.378545,-0.096750 -0.121900,-0.275556 -0.026534,0.119184 0.304656,0.043708 0.108648,-0.359507 -0.294795,-0.112865 -0.154452,0.151046 -0.348811,0.032201

Rhadinosuchinae_espejada 0.236826,0.158787 -0.277352,0.172569 0.236776,0.158796 0.363617,-0.098992 -0.085885,-0.238872 -0.039033,0.110198 0.282392,0.007281 0.143415,-0.307169 -0.320235,-0.124577 -0.163333,0.131285 -0.394887,0.035728

Asilisaurus_kongwe_1 0.233277,0.125927 -0.309152,0.179166 0.233721,0.125845 0.372913,-0.094040 -0.076138,-0.197311 -0.034461,0.078145 0.278740,-0.022515 0.239895,-0.273704 -0.336396,-0.095147 -0.178800,0.114468 -0.424748,0.057220

Sctuellosaurus_lawleri_UCMP_130 0.239904,0.067952 -0.318716,0.188384 0.240341,0.067872 0.372124,-0.016775 -0.066427,-0.216122 -0.036011,0.122310 0.310199,0.015039 0.164518,-0.287067 -0.319434,-0.133732 -0.176989,0.174830 -0.422857,0.025154

Effigia_okeeffeae_espejada 0.182345,0.178971 -0.220069,0.201566 0.183136,0.178826 0.351803,-0.068310 -0.082156,-0.351278 -0.015689,0.121270 0.289250,0.068606 0.119191,-0.418820 -0.337348,-0.114869 -0.130944,0.133773 -0.336045,0.070728

Fasolasuchus_tenax_PVL_3850 0.254265,0.200752 -0.216240,0.178573 0.254950,0.200207 0.279143,-0.020642 -0.122268,-0.346656 0.034200,0.091353 0.227556,0.078524 0.125122,-0.416139 -0.380212,-0.120138 -0.095266,0.128044 -0.346266,0.036589

Adeopapposaurus_mognai_PVSJ_610 0.283692,0.126786 -0.316474,0.145472 0.284640,0.126119 0.306844,-0.092344 -0.088678,-0.167994 -0.014837,0.079818 0.287588,-0.000718 0.237578,-0.251233 -0.342905,-0.097458 -0.171508,0.084448 -0.462425,0.039666

Aetosaurus_ferratus 0.221581,0.210551 -0.211015,0.152956 0.222382,0.210404 0.305571,-0.078519 -0.109133,-0.348687 0.003008,0.168966 0.287929,0.059898 0.041634,-0.414363 -0.343149,-0.146831 -0.110082,0.167737 -0.320190,-0.001651

Azendohsaurus_madagaskarensis 0.143080,0.192171 -0.310238,0.098427 0.316903,0.187905 0.417471,-0.153606 -0.108822,-0.275783 -0.098052,0.194124 0.375980,0.015468 -0.008518,-0.308799 -0.233346,-0.102606 -0.204642,0.160859 -0.286247,0.006301

Ceratosaurus_nasicornis_USNM_47 0.261498,0.187141 -0.270358,0.118992 0.261725,0.187099 0.303617,-0.120156 -0.110783,-0.181675 -0.001955,0.131460

0.243129,0.014938 0.174101,-0.380603 -0.333501,-0.117920 -0.137843,0.143533 -0.396768,-0.006226

Cryolophosaurus_ellioti 0.277272,0.186484 -0.280524,0.142789 0.277411,0.186458 0.319525,-0.139186 -0.083636,-0.189897 0.003472,0.098620 0.226214,0.015100 0.153475,-0.312798 -0.376143,-0.136180 -0.140973,0.130004 -0.389330,-0.011649

Dilophosaurus_wetherilli_UCMP_3 0.286929,0.184820 -0.289907,0.128567 0.287185,0.184773 0.305264,-0.128386 -0.100725,-0.177491 0.000879,0.124182 0.228293,0.001288 0.168975,-0.320763 -0.348184,-0.130305 -0.147831,0.138816 -0.394871,-0.014166

Eoraptor_lunensis_espejada 0.293512,0.136973 -0.311023,0.161697 0.293721,0.136935 0.327367,-0.098853 -0.078256,-0.178800 -0.018819,0.098488 0.241651,-0.000724 0.180803,-0.256737 -0.366649,-0.140979 -0.172378,0.125058 -0.409480,0.004823

Euparkeria_capensis_UMZC_T692 0.208910,0.130503 -0.297184,0.161471 0.208463,0.129220 0.398939,-0.113516 -0.095902,-0.245639 -0.044215,0.131360 0.325044,0.024742 0.145501,-0.296298 -0.298354,-0.108318 -0.175061,0.153447 -0.381771,0.032602

Herrerasaurus_ischigualastensis 0.267866,0.127600 -0.326118,0.187512 0.268520,0.127912 0.332130,-0.141010 -0.049158,-0.162452 -0.066208,0.115211 0.260220,-0.029024 0.246171,-0.254488 -0.328454,-0.125992 -0.209762,0.139441 -0.405494,0.029637

Jaklapallisaurus_assymetrica_IS 0.264736,0.142222 -0.309540,0.151551 0.264280,0.141095 0.334698,-0.097612 -0.076710,-0.207651 -0.019334,0.108951 0.265680,0.003303 0.185390,-0.283588 -0.345914,-0.109852 -0.168206,0.142320 -0.402301,0.020108

Lewisuchus_admixtus_MACN_Pv 0.226990,0.127344 -0.278375,0.192398 0.227016,0.127776 0.367466,-0.075854 -0.047081,-0.225587 -0.028929,0.083629 0.271638,-0.002525 0.210257,-0.279211 -0.386934,-0.107402 -0.164787,0.116797 -0.414807,0.058931

Liliensternus_liliensterni_MB_R 0.248183,0.156593 -0.282460,0.160307 0.248437,0.157003 0.340514,-0.121461 -0.066144,-0.178175 -0.027318,0.145170 0.237025,-0.012101 0.181550,-0.335737 -0.351342,-0.139489 -0.158521,0.153428 -0.392726,-0.001150

Mandasuchus_tanyauchen 0.259413,0.174613 -0.280897,0.147592 0.259414,0.174613 0.343364,-0.107083 -0.104694,-0.267950 -0.027807,0.140242 0.273198,0.019960 0.095138,-0.311609 -0.321149,-0.136955 -0.162648,0.156987 -0.350610,0.001346

Marasuchus_lilloensis 0.206915,0.154796 -0.238493,0.186270 0.207231,0.154738 0.418496,-0.078029 -0.103648,-0.260313 -0.027948,0.086034 0.262266,-0.009019 0.172851,-0.293661 -0.369736,-0.125641 -0.136124,0.126961 -0.392277,0.050311

Megapnosaurus_rhodesiensis_QR_6 0.206759,0.117267 -0.297386,0.155467 0.207035,0.117216 0.390752,-0.109596 -0.069623,-0.196448 -0.046037,0.167446 0.295361,0.002623 0.165794,-0.346097 -0.317395,-0.124726 -0.180069,0.202425 -0.366336,-0.002041

Nundasuchus_songeaensis 0.243699,0.193945 -0.265998,0.153038 0.243783,0.193930 0.343673,-0.139308 -0.100046,-0.265755 -0.014015,0.128816 0.280291,0.007031 0.113311,-0.285614 -0.327282,-0.138125 -0.142509,0.137017 -0.382579,0.014372

Parasuchus_hislopi_ISI_R_espeja 0.239130,0.183564 -0.274462,0.157392 0.238828,0.183682 0.364984,-0.112461 -0.084067,-0.290198 -0.026751,0.129070 0.291273,0.022400 0.076912,-0.303434 -0.323967,-0.124069 -0.158194,0.132259 -0.355371,0.025863

Pisanosaurus_mertii_PVL_2577 0.308017,0.120875 -0.296257,0.113480 0.307243,0.121200 0.288345,-0.055140 -0.107411,-0.171571 0.007744,0.108387 0.306925,0.030652 0.131195,-0.259524 -0.420042,-0.137937 -0.154203,0.122110 -0.405549,-0.043032

Plateosaurus_engelhardti_SMNS_1 0.310825,0.100117 -0.314663,0.151792 0.310467,0.100217 0.281367,-0.037420 -0.103589,-0.161789 -0.021664,0.085503 0.290684,0.027984 0.219330,-0.268029 -0.352106,-0.121236 -0.175515,0.111117 -0.446165,0.020856

Polesinesuchus_aurelioi 0.276099,0.183361 -0.280697,0.120245 0.276387,0.183308 0.351313,-0.103105 -0.105012,-0.253627 -0.008803,0.125561 0.313498,0.036346 0.017949,-0.274547 -0.329119,-0.143162 -0.151285,0.143519 -0.367763,-0.019188

Postosuchus_alisonae_espejada 0.265634,0.194667 -0.243561,0.143747 0.266061,0.194588 0.294109,-0.056303 -0.153312,-0.319314 0.014471,0.125097 0.269655,0.064638 0.091190,-0.360796 -0.339283,-0.132076 -0.120366,0.126186 -0.352169,0.016881

Postosuchus_kirkpatricki 0.256315,0.177575 -0.294528,0.134220 0.256461,0.177548 0.315074,-0.107739 -0.129788,-0.262756 -0.019763,0.154521 0.331138,0.041927 0.080824,-0.323516 -0.299858,-0.130330 -0.169120,0.141594 -0.339169,0.002201

Powellvenator_podocitus_PVL_441 0.275066,0.108858 -0.286325,0.134983 0.275058,0.108265 0.305819,-0.059007 -0.099041,-0.182153 -0.012398,0.152479 0.251830,0.013421 0.195155,-0.334218 -0.367202,-0.143923 -0.157904,0.195769 -0.395713,-0.031433

Proterosuchus_alexanderi_NMQR_1 0.179772,0.218878 -0.289475,0.088069 0.313349,0.198426 0.406080,-0.104975 -0.163362,-0.229980 -0.039667,0.113611 0.378130,0.026624 -0.012081,-0.311477 -0.285958,-0.101075 -0.159985,0.110679 -0.326469,-0.008340

Proterosuchus_sp_AMNH_2237_espe 0.198896,0.181735 -0.293065,0.075785 0.336764,0.176871 0.405097,-0.095601 -0.187042,-0.222107 -0.047813,0.126599 0.378406,0.039109 0.010896,-0.270493 -0.309479,-0.102276 -0.167915,0.103045 -0.326313,-0.018244

Rauisuchus_tiradentes_espejada 0.225296,0.147089 -0.253767,0.192490 0.225623,0.147029 0.381034,-0.050872 -0.096965,-0.313132 -0.018020,0.103281 0.308681,0.050912 0.066791,-0.302356 -0.344279,-0.154454 -0.136425,0.157576 -0.359990,0.027580

Riojasuchus_tenuisceps_PVL_3827 0.261402,0.246524 -0.200210,0.162268 0.261457,0.246432 0.343025,-0.091681 -0.135068,-0.366732 0.038462,0.114926 0.190366,0.040031 0.039828,-0.333099 -0.391348,-0.152435 -0.086432,0.106806 -0.337626,0.002967

Saturnalia_tupiniquim_MCP_3844_ 0.285573,0.163540 -0.300938,0.155912 0.285069,0.163692 0.304314,-0.128384 -0.070224,-0.204379 -0.025322,0.101306 0.246044,-0.003457 0.185613,-0.271843 -0.365558,-0.126410 -0.172200,0.123277 -0.394957,0.011743

Shansisuchus_shansisuchus 0.073846,0.108120 -0.289600,0.169218 0.329831,0.088342 0.403463,-0.070604 -0.070527,-0.192185 -0.110007,0.091274 0.373236,0.012203 0.195263,-0.317001 -0.325998,-0.081750 -0.174273,0.131215 -0.385585,0.054984

Shringasaurus 0.185949,0.200061 -0.293733,0.078259 0.339722,0.163874 0.373895,-0.114509 -0.140757,-0.233220 -0.065131,0.176005 0.373827,0.015938 0.005246,-0.295630 -0.293926,-0.115977 -0.179159,0.137250 -0.318473,-0.029550

Silesaurus_opolensis_ZPAL_Ab_II 0.228190,0.150970 -0.321049,0.157182 0.228662,0.150883 0.370854,-0.156986 -0.058644,-0.173646 -0.053593,0.131672 0.263396,-0.031874 0.223197,-0.323029 -0.284098,-0.087429 -0.190140,0.144187 -0.407756,0.027547

Smilosuchus_gregorii_USNM_18313 0.289977,0.125859 -0.375576,0.132200 0.291128,0.125495 0.382613,-0.083947 -0.075951,-0.191554 -0.044133,0.103034 0.308336,0.006145 0.091257,-0.235527 -0.277313,-0.093588 -0.217746,0.128367 -0.398245,0.017916

Tawa_hallae_GR_242_espejada 0.220402,0.147525 -0.274185,0.189045 0.219761,0.146297 0.348202,-0.141954 -0.067399,-0.202045 -0.037577,0.097237 0.238765,-0.015788 0.277191,-0.300612 -0.355641,-0.115799 -0.154536,0.146751 -0.421031,0.048660

Vancleavea_campi 0.098081,0.235642 -0.267921,0.127575 0.301706,0.129644 0.366945,-0.175257 -0.081803,-0.211080 -0.093131,0.193568 0.341760,-0.021911 0.151489,-0.343617 -0.283282,-0.100786 -0.162746,0.158389 -0.358645,0.009217

Pamelaria_dolichotrachela_ISIR_ 0.136529,0.218546 -0.265453,0.137665 0.320318,0.186925 0.383908,-0.094976 -0.073590,-0.301692 -0.057982,0.143446 0.361899,0.029673 -0.020135,-0.326346 -0.296651,-0.113425 -0.144731,0.121086 -0.333133,0.023387

;

tread

(((Shansisuchus_shansisuchus (Vancleavea_campi (Rhadinosuchinae_espejada (Euparkeria_capensis_UMZC_T692 (((Marasuchus_lilloensis ((((Eoraptor_lunensis_espejada (Saturnalia_tupiniquim_MCP_3844_ (Jaklapallisaurus_assymetrica_IS (Adeopapposaurus_mognai_PVSJ_610 Plateosaurus_engelhardti_SMNS_1))))(Herrerasaurus_ischigualastensis (Tawa_hallae_GR_242_espejada ((Liliensternus_liliensterni_MB_R (Cryolophosaurus_ellioti (Ceratosaurus_nasicornis_USNM_47 Dilophosaurus_wetherilli_UCMP_3)))(Megapnosaurus_rhodesiensis_QR_6 Powellvenator_podocitus_PVL_441)))))(Pisanosaurus_mertii_PVL_2577 Sctuellosaurus_lawleri_UCMP_130))(Lewisuchus_admixtus_MACN_Pv (Asilisaurus_kongwe_1 Silesaurus_opolensis_ZPAL_Ab_II))))(Riojasuchus_tenuisceps_PVL_3827 ((Aetosaurus_ferratus Polesinesuchus_aurelioi)(Gracilisuchus_stipanicicorum_PV (Nundasuchus_songeaensis (Effigia_okeeffeae_espejada (Mandasuchus_tanyauchen (Fasolasuchus_tenax_PVL_3850 (Rauisuchus_tiradentes_espejada (Postosuchus_alisonae_espejada Postosuchus_kirkpatricki))))))))))(Parasuchus_hislopi_ISI_R_espeja (Pseudopalatus_UCMP_27235_espeja Smilosuchus_gregorii_USNM_18313)))))))(Proterosuchus_alexanderi_NMQR_1 Proterosuchus_sp_AMNH_2237_espe))(Pamelaria_dolichotrachela_ISIR_ (Azendohsaurus_madagaskarensis Shringasaurus)))

;

proc/;

ix) – Coordinates for terminal taxa.

LM=11

0.283043320026586 0.141385018723818

-0.283378017250646 0.128386975432158

0.282793779361182 0.140530623098962

0.316839357168764 -0.0372877455640155

-0.148063082220074 -0.242964775675276

-0.0212325342687737 0.114424365410904

0.312745506869908 0.0558648260441466

0.104251924194345 -0.327534516013807

-0.336894976220799 -0.107745417916454

-0.163816087707023 0.124595822956981

-0.367693830199856 0.0132503853072696

ID=Gracilisuchus_stipanicicorum_PVL_4597

LM=11

0.214559446020188 0.168809134423451

-0.27309662547295 0.163342178087267

0.214831959835925 0.168756604739999

0.377897800519534 -0.100463126899683

-0.124598372970745 -0.274533425518697

-0.025415077591887 0.119535190706343

0.305346545582405 0.040789873820117

0.105225738066983 -0.360730880613964

-0.296110243415682 -0.110127373736629

-0.153196997522427 0.152622196604037

-0.348870516014593 0.0348148952973444

ID=Pseudopalatus_UCMP_27235_espejada

LM=11

0.238241257545245 0.156922087819842

-0.276065973751885 0.174914681409143

0.238193745233404 0.156931246279182

0.362966352138838 -0.102015229820143

-0.0878911322033147 -0.238283455978066

-0.0381423092956447 0.110574871696865

0.282578989172759 0.0049765538918869

0.140971675399996 -0.308498882310788

-0.321415089895077 -0.122009443463829

-0.162289100456113 0.132680409389901

-0.394885385826191 0.0381914788707943

ID=Rhadinosuchinae_espejada

LM=11

0.234254203582144 0.12457018615034

-0.308305559203368 0.181256443914267

0.234895739964471 0.124446523784836

0.372670113523696 -0.0965642146741616

-0.0774657895907667 -0.196967589208504

-0.0339877706541911 0.0784905789940224

0.278988173345503 -0.0243224593003051

0.23806697532313 -0.275424053399775

-0.337274323197264 -0.0930768067913725

-0.178059469350875 0.115643102682564

-0.424811614106895 0.0596145113847928

ID=Asilisaurus_kongwe_1

LM=11

0.240391759875902 0.0661544194288113

-0.317364826933044 0.190771150768219

0.240990085915401 0.0660390862763583

0.372072780142108 -0.0196298561484685

-0.0680398816018478 -0.215579917213488

-0.0350933693724508 0.122604334557625

0.310458662932379 0.0126692356600247

0.162190490723129 -0.288238025201341

-0.320441652838602 -0.131316506721025

-0.175474891366061 0.176087574722975

-0.422762004888049 0.0270775919341664

ID=Sctuellosaurus_lawleri_UCMP_130580_espejada

LM=11

0.184614292812864 0.17753383384082

-0.21881686035276 0.204048040391044

0.185789121858831 0.177307374137381

0.35203848912396 -0.0721257056462512

-0.0858590856312322 -0.35088688530956

-0.0145506325814323 0.121738825428422

0.290870891315058 0.0656988176914307

0.114968195009219 -0.420546339835409

-0.339220861242382 -0.111754322143415

-0.129791395485764 0.135242125988251

-0.33666631903405 0.0732180506479829

ID=Effigia_okeeffeae_espejada

LM=11

0.256974708955652 0.198420027726444

-0.215191386154055 0.1812250578946

0.25815186071974 0.197773050339641

0.279996067796229 -0.0240119870626706

-0.126240578253106 -0.345789333690189

0.0350971444669338 0.0913331459535627

0.229526736945361 0.0760391535144876

0.120497279446815 -0.418068222128799

-0.382326265684677 -0.116293004893774

-0.0949449544051182 0.129563437933693

-0.347797103519283 0.0389897516400385

ID=Fasolasuchus_tenax_PVL_3850

LM=11

0.284509510432721 0.124919437531451

-0.315622865159071 0.147557029519391

0.285693743492133 0.124197976502996

0.306351055938176 -0.0944524226760188

-0.0897698007812324 -0.167351022351497

-0.0143174251982879 0.0799544831624356

0.287809622131441 -0.00267154163102081

0.235672979216419 -0.252722127936363

-0.343559841881935 -0.095173989098362

-0.171101670085403 0.0855842138009946

-0.462348118261823 0.0423023087911748

ID=Adeopapposaurus_mognai_PVSJ_610

LM=11

0.224188106587032 0.208188566709574

-0.209678414473499 0.155470229233841

0.225326158306654 0.20796919619281

0.305154299903289 -0.0821895431003957

-0.113206171232631 -0.347572390930868

0.00491168424448041 0.169083310577755

0.289182416713813 0.0565618037996819

0.0366964770784761 -0.414979210502981

-0.345150176155216 -0.143004005308207

-0.109154973846147 0.169023745180783

-0.321531286751353 0.000284292858464754

ID=Aetosaurus_ferratus

LM=11

0.156789257543866 0.174669665938374

-0.250707280742831 0.102740569714173

0.314992456181009 0.165469570587216

0.394887516304182 -0.144742921270965

-0.0811886350839873 -0.237881458290587

-0.0584764545609789 0.184331848916759

0.363278702234695 0.00875950264396274

0.00511808876402459 -0.269457467230452

-0.29406859023563 -0.133684438320407

-0.142763877357615 0.161962013112162

-0.391788251870582 -0.0206098613317851

ID=Azendohsaurus_central

LM=11

0.263599019895341 0.184729390272825

-0.269532281111847 0.121827533711488

0.263955491534706 0.184660676903835

0.30285304559882 -0.123296657975608

-0.112677650921695 -0.180810361266156

-0.00068591679155414 0.131625472365588

0.243685155671539 0.0125311223576821

0.170369958026646 -0.382625038687591

-0.335032631163858 -0.114741238241918

-0.137450382677339 0.145004855927302

-0.397794214507952 -0.004084520959463

ID=Ceratosaurus_nasicornis_USNM_4735

LM=11

0.279053191669102 0.184056522750951

-0.279388374236471 0.145383158466294

0.279263797309032 0.184015926469368

0.318453218754602 -0.14212722816583

-0.0853873813847773 -0.189235740476958

0.00434631039745871 0.0986525475603199

0.226536074731092 0.013057495075723

0.150654184624536 -0.314298660759173

-0.377519861873143 -0.132836701422032

-0.140810389119459 0.131484142475149

-0.390237714059664 -0.00983370263018086

ID=Cryolophosaurus_ellioti

LM=11

0.288698553119817 0.182333676194767

-0.288947432898086 0.131239180792101

0.289071681483445 0.182261752080478

0.30434262644618 -0.131202654546557

-0.102357042364616 -0.176686919331727

0.00197496787498044 0.124251526880045

0.228552008267412 -0.00076679839909582

0.166062627664622 -0.322400528782852

-0.349528087837711 -0.12723805352458

-0.147729332488953 0.140225861035973

-0.395629591682843 -0.0122265332187898

ID=Dilophosaurus_wetherilli_UCMP_37302

LM=11

0.294536110563005 0.134772673820644

-0.309846844643273 0.164032712675184

0.294823944823577 0.134717190972724

0.326667707511239 -0.101339644014713

-0.0795951550283558 -0.178198461253132

-0.0180820264055717 0.0986438828932912

0.241724349134665 -0.0025537673089112

0.17879598517133 -0.258073174934187

-0.367715953289223 -0.13821890930164

-0.172166408271741 0.126508527970086

-0.409739827063576 0.00661403153062013

ID=Eoraptor_lunensis_espejada

LM=11

0.209944925189098 0.128844209747278

-0.295925530092311 0.163835173179038

0.209549081926297 0.127552480437482

0.398062273578074 -0.116714383402346

-0.0978550756101854 -0.244859574613729

-0.0431690842479477 0.131721523531927

0.325297312607829 0.0221390680204028

0.143078964573816 -0.297434166511773

-0.299220050138378 -0.105938646435214

-0.173736176013777 0.154821065601723

-0.381552279973335 0.0350898738969892

ID=Euparkeria_capensis_UMZC_T692

LM=11

0.268701697057445 0.126093876565054

-0.325219501216969 0.189493369104467

0.26957325837487 0.126358762019817

0.331539534682144 -0.143081045094482

-0.0501439309163565 -0.162213073112769

-0.0655425892060765 0.115687131383294

0.260361418147303 -0.0305954487491465

0.244514993370524 -0.256001657359198

-0.329347789338326 -0.124087637259588

-0.20929939996758 0.140794830899596

-0.405601807252552 0.0313701117713737

ID=Herrerasaurus_ischigualastensis_PVSJ_373

LM=11

0.265925682980935 0.140250868204656

-0.308508239724929 0.153997200168498

0.265518614106616 0.139115662537717

0.334111974911455 -0.100247609939442

-0.0783486334009823 -0.207146697670161

-0.0185070554218497 0.109157961371506

0.265868951151766 0.00125322549493067

0.183211742844909 -0.285130350354007

-0.346899878198448 -0.107232123090575

-0.167677186994653 0.143745842216929

-0.402480780424875 0.0221637293500638

ID=Jaklapallisaurus_assymetrica_ISI_R274_espejada

LM=11

0.228105386328871 0.12589472520371

-0.277329258107027 0.194505892162725

0.228276661411608 0.126298447970624

0.367313665888615 -0.0785899326011776

-0.0487654628521406 -0.225449129515533

-0.028366079770779 0.0839686462578793

0.272001635587661 -0.00445648103372198

0.208284139687613 -0.280954241806307

-0.388011368531548 -0.10476403026252

-0.164074570446583 0.118059733395493

-0.415212100611699 0.0607949467056814

ID=Lewisuchus_admixtus_MACN_Pv

LM=11

0.249720489004859 0.154606263016165

-0.281344157426057 0.162851831890934

0.250045195047244 0.155000507231801

0.339789220782267 -0.124473087904834

-0.0677501002238135 -0.177799763425267

-0.0260978453675189 0.14551368063511

0.23720743798896 -0.0141239381205353

0.178740765024048 -0.337538991273457

-0.352818964479268 -0.136576880086964

-0.158146573373663 0.154952203585928

-0.393510450879446 0.000417214980239613

ID=Liliensternus_liliensterni_MB_R2175_espejada

LM=11

0.261037476539125 0.172255669011347

-0.279579166288525 0.15018088736762

0.261045050104418 0.172254209133115

0.342425023152768 -0.110238241934551
-0.107157959583892 -0.267016407485048
-0.0265258260804828 0.140510761863953
0.273416386705274 0.0174600340651994
0.0922990355314393 -0.312515399626832
-0.322442116049736 -0.134027423492359
-0.161965917250666 0.158519686150869
-0.350811210354252 0.00344405887874185
ID=Mandasuchus_tanyauchen

LM=11
0.208406511932891 0.153325551985246
-0.237372200324328 0.188337523413619
0.208885950210415 0.153233135603034
0.41831922289908 -0.0815375231050036
-0.105834713620298 -0.259708993917015
-0.0272932028553094 0.0864148459506533
0.262638188092566 -0.0111263381372341
0.1705101349117 -0.295342264686048
-0.371115262010966 -0.122801617371195
-0.135218200188719 0.128146330521661
-0.392884384633378 0.0522615716332873
ID=Marasuchus_lilloensis

LM=11
0.207886943135621 0.115555607122688
-0.296221715413039 0.158125464541768
0.208287541411532 0.115478387916139
0.390020887716453 -0.11308380266494
-0.0713743615151024 -0.195934742167863

-0.0445923944523938 0.167924226040355

0.295630006372858 3.70643417423044e-05

0.162731722868778 -0.347652698589933

-0.318651240650481 -0.122015916880854

-0.178816676920778 0.204032721850115

-0.366754644181259 -0.000736139930288589

ID=Megapnosaurus_rhodesiensis_QR_691_espejada

LM=11

0.245355659699008 0.191906982191842

-0.264751160467245 0.155294380888702

0.245475835653968 0.191883817109898

0.342548882117946 -0.142224945158421

-0.102295151618327 -0.264925450602277

-0.0129325050527548 0.128951022489707

0.280410834867103 0.00466283300744726

0.110883590695776 -0.286588699773715

-0.328481744543588 -0.135374288447803

-0.141710207509314 0.138263902853479

-0.382712564124066 0.0166963939766757

ID=Nundasuchus_songeaensis

LM=11

0.240724626193551 0.181429929097756

-0.27305074730675 0.159800858168079

0.240450819380929 0.181545040937947

0.363961986729174 -0.115687997774232

-0.0866229402425918 -0.289417436640346

-0.0256069029500676 0.129296697905327

0.291465578660223 0.0198141675783368

0.0741835182637552 -0.304066142916908

-0.325028019563661 -0.121187065976016

-0.157297258530478 0.133674814414098

-0.355237147263016 0.028350494641835

ID=Parasuchus_hislopi_ISI_R_espejada

LM=11

0.308839536208559 0.117818641473607

-0.294889153330281 0.116044964654516

0.308099817655813 0.118144962972871

0.287440248829664 -0.0577185118171152

-0.108877088387234 -0.170271618281456

0.00877975385904528 0.108173846536624

0.306914730524864 0.0277658960406142

0.12855957353252 -0.260336841867513

-0.420915545504749 -0.133881957251537

-0.153871021711007 0.12339069695241

-0.406673321225269 -0.0415485055427411

ID=Pisanosaurus_mertii_PVL_2577

LM=11

0.311551334400688 0.0977628123015004

-0.313625583175449 0.154166556487193

0.311456516862761 0.0978134365486518

0.28120997574758 -0.0396359766755653

-0.10479020376444 -0.160934674689327

-0.0210239732241261 0.0857061490790574

0.291138529765422 0.0257215001494587

0.217028608548142 -0.269596442138162

-0.353030577499127 -0.118542627389253

-0.175100295923796 0.112512010386685

-0.446320324279355 0.0230534705733088

ID=Plateosaurus_engelhardti_SMNS_13200

LM=11

0.277491494695341 0.180398126736864

-0.279099770669567 0.122734254821254

0.277820912058736 0.180334628342201

0.349770323390459 -0.106404492972689

-0.107322256580651 -0.252189048023716

-0.00753450490822289 0.125456000236556

0.313456108404189 0.0332349200046159

0.0149681982471759 -0.274166782138032

-0.329973279223529 -0.139743082440583

-0.150110057484348 0.144752867205532

-0.367641287366848 -0.0169580962106276

ID=Polesinesuchus_aurelioi

LM=11

0.267901213230152 0.192044846188067

-0.242416172910781 0.14644100652352

0.268528786888894 0.19192387527308

0.293957309126185 -0.0595402054945341

-0.156754988242669 -0.317887123234678

0.0157512752612641 0.125090397648048

0.270790678408334 0.0617891903390634

0.0873582379256804 -0.361991161000684

-0.341009518173627 -0.128622835328965

-0.120065693463429 0.127635576051925

-0.352865134953294 0.0194850006581565

ID=Postosuchus_alisonae_espejada

LM=11

0.257924870472039 0.175063560172762

-0.293164159995528 0.136984227194746

0.258112293258781 0.175027432611183

0.313941660063627 -0.110735755556198

-0.13226462819993 -0.261413147735327

-0.0182742126044731 0.154671567539394

0.331480868042404 0.0387370282606274

0.0776296373453362 -0.324155633067363

-0.300990124067751 -0.127413383608932

-0.168204146134772 0.14312684329543

-0.339066280103829 0.00465444679642661

ID=Postosuchus_kirkpatricki

LM=11

0.276242175731248 0.106463908440095

-0.285358759213644 0.137730697908844

0.276361299773116 0.105845744582195

0.305611191406803 -0.0619315379816698

-0.100783656411959 -0.181405610088609

-0.0110196409505493 0.152705675753735

0.25228848027017 0.0110984112966535

0.192073814038829 -0.336221615918752

-0.368794621631075 -0.140633148050045

-0.15731257116353 0.197258530976013

-0.397147987606473 -0.0303812784281472

ID=Powellvenator_podocitus_PVL_4414_espejada

LM=11

0.211760071797736 0.16952190599581

-0.28171720418317 0.126949436415579

0.319083465627803 0.148563013272939

0.383516549091422 -0.0980161180341317

-0.077894026688809 -0.179450793387316

-0.0389531133433303 0.0906794771808599

0.365702667369686 0.00848207723706565

0.0399516846765955 -0.24961386283739

-0.332108014049381 -0.122877298352342

-0.153068880692525 0.0800539350691304

-0.419528004760802 0.0125978454955946

ID=P_alexanderi_central

LM=11

0.225962653641279 0.138122881034819

-0.345015316840781 0.132338451201204

0.337349802426095 0.130509394610775

0.384896863050552 -0.0901515031177805

-0.0947256768074775 -0.17596082435691

-0.0441268102951648 0.0812324962430632

0.367282384526312 0.0188133330945274

0.062554875611319 -0.219763817404812

-0.315446384570707 -0.102946800359871

-0.197635433296833 0.0754683982026265

-0.388371908362547 0.0264279415878391

ID=P_sp_central

LM=11

0.226668061130466 0.145041754525586

-0.252084249641628 0.194833713472379

0.227122740620534 0.144954110640006

0.380667655491348 -0.0544366658616306

-0.0998568176263809 -0.31223353688637

-0.0170766491832288 0.103480545274636

0.309301666028621 0.0480430683976214

0.0639005575024324 -0.30295611634145

-0.34574762517609 -0.151288355060761

-0.134784001854226 0.158742197322786

-0.360191548413632 0.0296408349761293

ID=Rauisuchus_tiradentes_espejada

LM=11

0.264171510840754 0.243955107728114

-0.198958059211707 0.16444679971783

0.264651416401528 0.243782671384867

0.342577002286338 -0.0954736683736695

-0.138982857811323 -0.365421376282943

0.0396212910195989 0.114681135935541

0.191430989042766 0.0380032321951583

0.0360702988136582 -0.33361597097866

-0.393257544599459 -0.14842375652005

-0.0866679444844871 0.108013295581585

-0.33892899459338 0.00527741639031255

ID=Riojasuchus_tenuisceps_PVL_3827

LM=11

0.286898562398406 0.161456848240563

-0.299902662871779 0.158240039973232

0.286458103037647 0.161600680600583

0.303516832762465 -0.130733218537482

-0.0718022941075127 -0.203936601684118

-0.0245713424321545 0.101555817668633

0.246186610667994 -0.00531348496074523

0.183567466332057 -0.273354153673209

-0.366650733518934 -0.123700668661972

-0.172048827596844 0.124772769392864

-0.395302014787433 0.013676239245055

ID=Saturnalia_tupiniquim_MCP_3844_PV_espejada

LM=11

0.0740989538531368 0.107123283861941

-0.28760502964361 0.171246576630817

0.330223983432495 0.0850696249223875

0.402136541894987 -0.0738989959657732

-0.0718594958717712 -0.190876898340008

-0.109175020549815 0.0919586314768423

0.372940545394938 0.00890337603902496

0.191830589137514 -0.317684985100524

-0.325819982532575 -0.0786576057594588

-0.177413477826891 0.13441134086512

-0.384536801804813 0.0569554302009621

ID=Shansisuchus_shansisuchus

LM=11

0.224007451389044 0.171166692673386

-0.331298677096077 0.0955691011604325

0.352798804190957 0.129402654484806

0.362365181431057 -0.109896210018305

-0.0840658762402606 -0.175015438150693

-0.0557040047095765 0.150636384661078

0.371589803946426 0.0010900514942047

0.0354936530430806 -0.238043411475203

-0.301274136577483 -0.104127852862553

-0.185212092352633 0.101231315528681

-0.385512087432542 -0.00798464787396072

ID=Shringasaurus_central

LM=11

0.229375599580678 0.149499571004079

-0.320223020681847 0.159501326316073

0.230050627677945 0.149369452785394

0.370073790572797 -0.159685121316098

-0.0598982291138082 -0.173349234548396

-0.0526983316756724 0.132152777767857

0.263533267662015 -0.0337280392382481

0.220848983768692 -0.324713883456452

-0.284927656997404 -0.0855254779443382

-0.189352987577268 0.145588497422427

-0.407872057866977 0.030109342532715

ID=Silesaurus_opolensis_ZPAL_Ab_III_361

LM=11

0.290360783277961 0.123427568191492

-0.374049641763896 0.134571134113419

0.291523683310679 0.123052362317028

0.381288762503736 -0.0865115432194978

-0.0771094626384223 -0.190481758769447

-0.0433351005298792 0.103098419220737

0.307822361316863 0.00387599955137134

0.0892049366004485 -0.235476499794577

-0.277268645845377 -0.0913339441647195

-0.21638203795314 0.129614500204321

-0.397567197365713 0.0212489296776634

ID=Smilosuchus_gregorii_USNM_18313

LM=11

0.221821216765987 0.146390440769455

-0.273578335483934 0.191143597398484

0.221449606612562 0.145111249801028

0.348042771500652 -0.144525134078344

-0.0689137286139074 -0.202002699257878

-0.0369910144890834 0.0977594375513195

0.239431253112034 -0.0173802273823301

0.275225190826828 -0.302979236936594

-0.357063020417693 -0.113657844146228

-0.154002793344903 0.14805914514765

-0.421865462767927 0.0503832159548311

ID=Tawa_hallae_GR_242_espejada

LM=11

0.0997265400741141 0.234548609774524

-0.266473867048534 0.129634499559872

0.302648189899764 0.126704817585494

0.364957313105757 -0.178381148943317

-0.0834524188185492 -0.209902765731382

-0.0913935984713444 0.19422357222371

0.341345245034758 -0.0249812849676059

0.147976067621627 -0.344297723235197

-0.283609948895064 -0.0981093698818802

-0.164775334528906 0.158137723887833

-0.358138034817907 0.0117862503640172

ID=Vancleavea_campi

LM=11

0.190244916255264 0.170149779892659

-0.36010809018044 0.146172492856054

0.330211920020622 0.12675032889001

0.351365093988869 -0.0986188650991898

-0.025001084545358 -0.21361144538319

0.0316034020606404 0.130928904333608

0.34684291970159 0.000350089693261712

0.0138138914039818 -0.237466420674096

-0.284714177922299 -0.132504197215744

-0.141766806167768 0.135362791990976

-0.438050569194609 -0.00201811393101118

ID=P_dolichotrachela

LM=11

0.138022374638997 0.216596224478366

-0.26319327393044 0.139927350497212

0.321665452346805 0.182964586667545

0.381867620157235 -0.0987917494044328

-0.0764296295021721 -0.300048764294125

-0.0563811031321093 0.143692123400304

0.361508628403607 0.0257280781125897

-0.0243128923915584 -0.324925063999596

-0.296731407687878 -0.109889488683773

-0.147085962865403 0.122014716771647

-0.331830539530998 0.0265032119504003

ID=P_dolichotrachela_sin_central

LM=11

0.187223562806396 0.197524194539709

-0.292013748530513 0.0808370407321722

0.340726777616848 0.159652779648436

0.37153511951201 -0.118216871304958

-0.142869610223195 -0.230817657845131

-0.0631553515586303 0.176298497340757

0.373172015877222 0.0118373914387255

0.00140761005597868 -0.294458754047039

-0.293982932898835 -0.112416723829592

-0.180499882662358 0.137212777912897

-0.317594292237422 -0.0268370989655567

ID=Shringasaurus_sin_central

LM=11

0.181337113994946 0.21632426616715

-0.287622411251163 0.0907220574798656

0.31484802989356 0.194331853083767

0.403780115739545 -0.109225516743617

-0.165533203100278 -0.227237426596715

-0.0383166944682613 0.113653151134331

0.377631415584508 0.0222933637469109

-0.0162796681912855 -0.310074519639455

-0.285886546811234 -0.097469008107099

-0.161468420046816 0.111612030607616

-0.325366817514995 -0.00516665833503235

ID=P_alexanderi_sin_central

LM=11

0.199974326878308 0.178955044200987

-0.291293641877153 0.078417690296878

0.337928274404933 0.172523625750977

0.402835181580589 -0.0997707185188801

-0.189080661241752 -0.219076443312182

-0.0463235761183065 0.126699064227447

0.37796903874336 0.0348180596137224

0.00718114375164547 -0.269298948159746

-0.309342021052158 -0.0984146832493477

-0.169952448340271 0.10357694882771

-0.325288732447848 -0.0152439552208541

ID=P_sp_sin_central

LM=11

0.144109394381055 0.190141849032344

-0.308367097418077 0.100994391828442

0.31819644997325 0.183994857455915

0.414750375258516 -0.15764053591739

-0.111238124469821 -0.273662255500265

-0.0959552151550517 0.194734423002531

0.375419809244157 0.0114592448836243

-0.0125919065667853 -0.307306705041012

-0.233132994401082 -0.0997759853046689

-0.20607949303724 0.16069076869717

-0.285100938193316 0.00935905371791677

ID=Azendoh_sin_central

x) – References

1. Schoch, R. R. Osteology of the small archosaur *Aetosaurus* from the Upper Triassic of Germany. *Neues Jahrb. Geol. P-A.* **246**, 1–35 (2007).

2. Nesbitt, S. J., Langer, M. C. & Ezcurra, M. D. The anatomy of *Asilisaurus kongwe*, a dinosauriform from the Lifua Member of the Manda Beds (~Middle Triassic) of Africa. *Anat. Rer*. (In press).

3. Nesbitt, S. J. et al. Postcranial anatomy and relationships of *Azendohsaurus madagaskarensis*. *B. Am. Mus. Nat. Hist.* **398**, 1–126 (2015).

4. Smith, N. D., Makovicky, P. J., Hammer, W. R. & Currie, P. J. Osteology of *Cryolophosaurus ellioti* (Dinosauria: Theropoda) from the Early Jurassic of Antarctica and implications for early theropod evolution. *Zool. J. Linn. Soc.* **151**, 377–421 (2007).

5. Nesbitt, S. The anatomy of *Effigia okeeffeae* (Archosauria, Suchia), theropod-like convergence, and the distribution of related taxa. *B. Am. Mus. Nat. Hist*. **2007**, 1–85 (2007).

6. Sereno, P. C., Martínez, R. N. & Alcober, O. A. Osteology of *Eoraptor lunensis* (Dinosauria, Sauropodomorpha). *J. Vertebr. Paleontol.* **32**, 83–179 (2012).

7. Butler, R. J., Nesbitt, S. J., Charig, A. J., Gower, D. J. & Barrett, P. M. *Mandasuchus tanyauchen*, gen. et sp. nov., a pseudosuchian archosaur from the Manda Beds (? Middle Triassic) of Tanzania. *J. Vertebr. Paleontol.* **37**, 96–121 (2017).

8. Sereno, P. C. & Arcucci, A. B. Dinosaurian precursors from the Middle Triassic of Argentina: *Marasuchus lilloensis*, gen. nov. *J. Vertebr. Paleontol.* **14**, 53–73 (1994).

9. Nesbitt, S. J., Sidor, C. A., Angielczyk, K. D., Smith, R. M. & Tsuji, L. A. A new archosaur from the Manda beds (Anisian, Middle Triassic) of southern Tanzania and its implications for character state optimizations at Archosauria and Pseudosuchia. *J. Vertebr. Paleontol.* **34**, 1357–1382 (2014).

10. Roberto-Da-Silva, L., Desojo, J. B., Cabreira, S. F., Aires, A. S., Mueller, R. T., Pacheco, C. P. & Dias-Da-Silva, S. A new aetosaur from the Upper Triassic of the Santa Maria Formation, southern Brazil. *Zootaxa* **3764**, 240–278 (2014).

11. Peyer, K., Carter, J. G., Sues, H. D., Novak, S. E. & Olsen, P. E. A new suchian archosaur from the Upper Triassic of North Carolina. *J. Vertebr. Paleontol.* **28**, 363–381 (2008).

12. Weinbaum, J. C. in *Anatomy, Phylogeny and Palaeobiology of Early Archosaurs and their Kin* (eds. Nesbitt, S. J., Desojo, J. B. & Irmis, R. B.) 525–553 (Geological Society, Special Publications, London, 2013).

13. Nesbitt, S. J. The early evolution of archosaurs: relationships and the origin of major clades. *B. Am. Mus. Nat. Hist.* **352**, 1–292 (2011).

14. Lautenschlager, S. & Rauhut, O. W. Osteology of *Rauisuchus tiradentes* from the Late Triassic (Carnian) Santa Maria Formation of Brazil, and its implications for rauisuchid anatomy and phylogeny. *Zool. J. Linn. Soc.* **173**, 55–91 (2015).

15. Gower, D. J. The tarsus of erythrosuchid archosaurs, and implications for early diapsid phylogeny. *Zool. J. Linn. Soc.* **116**, 347–375 (1996).

16. Nesbitt, S. J., Stocker, M. R., Small, B. J. & Downs, A. The osteology and relationships of *Vancleavea campi* (Reptilia: Archosauriformes). *Zool. J. Linn. Soc*. **157**, 814–864 (2009).

17. Bookstein, F. L. Landmark methods for forms without landmarks: morphometrics of group differences in outline shape. *Med. Image Anal.* **1,** 225–43 (1997).

18. Goloboff, P. A. & Catalano, S. A. TNT version 1.5, including a full implementation of phylogenetic morphometrics. *Cladistics* **32**, 221–238 (2016).

19. Ezcurra, M. D. The phylogenetic relationships of basal archosauromorphs, with an emphasis on the systematics of proterosuchian archosauriforms. *PeerJ* **4**, e1778 (2016).

20. Ezcurra, M. D. et al. Deep faunistic turnovers preceded the rise of dinosaurs in southwestern Pangaea. *Nat. Ecol. Evol.* **1**, 1477 (2017).

21. Sengupta, S., Ezcurra, M. D. & Bandyopadhyay, S. A new horned and long-necked herbivorous stem-archosaur from the Middle Triassic of India. *Sci. Rep.* **7**, 8366 (2017).

22. Nesbitt, S. & Desojo, J. B. The osteology and phylogenetic position of *Luperosuchus fractus* (Archosauria: Loricata) from the latest Middle Triassic or earliest Late Triassic of Argentina. *Ameghiniana* **54**, 261–283 (2017).

23. Ezcurra, M. D. A new Early Coelophysoid neotheropod from the Late Triassic of northwestern Argentina. *Ameghiniana* **54**, 506–539 (2017).

24. Müller, R. T. et al. Early evolution of sauropodomorphs: anatomy and phylogenetic relationships of a remarkably well-preserved dinosaur from the Upper Triassic of southern Brazil. *Zool. J. Linn. Soc.* **184**, 1187–1248 (2018).
